# Exploring the Conformational Changes Induced by Nanosecond Pulsed Electric Fields on the Voltage Sensing Domain of a Ca^2+^ Channel

**DOI:** 10.3390/membranes11070473

**Published:** 2021-06-26

**Authors:** Alvaro R. Ruiz-Fernández, Leonardo Campos, Felipe Villanelo, Sebastian E. Gutiérrez-Maldonado, Tomas Perez-Acle

**Affiliations:** 1 Computational Biology Lab, Fundación Ciencia & Vida, Zañartu 1482, Ñuñoa, Santiago 7780272, Chile; leocampos@dlab.cl (L.C.); felipe@dlab.cl (F.V.); sebastian@dlab.cl (S.E.G.-M.); 2 Facultad de Ingeniería y Tecnología, Universidad San Sebastián, Santiago 8420524, Chile; 3 Centro Interdisciplinario de Neurociencia de Valparaíso, Universidad de Valparaíso, Valparaíso 2360102, Chile

**Keywords:** nsPEF, NPS, nanopores, ionic channels, VSD, cholesterol

## Abstract

Nanosecond Pulsed Electric Field (nsPEF or Nano Pulsed Stimulation, NPS) is a technology that delivers a series of pulses of high-voltage electric fields during a short period of time, in the order of nanoseconds. The main consequence of nsPEF upon cells is the formation of nanopores, which is followed by the gating of ionic channels. Literature is conclusive in that the physiological mechanisms governing ion channel gating occur in the order of milliseconds. Hence, understanding how these channels can be activated by a nsPEF would be an important step in order to conciliate fundamental biophysical knowledge with improved nsPEF applications. To get insights on both the kinetics and thermodynamics of ion channel gating induced by nsPEF, in this work, we simulated the Voltage Sensing Domain (VSD) of a voltage-gated Ca${}^{2+}$ channel, inserted in phospholipidic membranes with different concentrations of cholesterol. We studied the conformational changes of the VSD under a nsPEF mimicked by the application of a continuous electric field lasting 50 ns with different intensities as an approach to reveal novel mechanisms leading to ion channel gating in such short timescales. Our results show that using a membrane with high cholesterol content, under an nsPEF of 50 ns and $\vec{E}$ = 0.2 V/nm, the VSD undergoes major conformational changes. As a whole, our work supports the notion that membrane composition may act as an allosteric regulator, specifically cholesterol content, which is fundamental for the response of the VSD to an external electric field. Moreover, changes on the VSD structure suggest that the gating of voltage-gated Ca${}^{2+}$ channels by a nsPEF may be due to major conformational changes elicited in response to the external electric field. Finally, the VSD/cholesterol-bilayer under an nsPEF of 50 ns and $\vec{E}$ = 0.2 V/nm elicits a pore formation across the VSD suggesting a new non-reported effect of nsPEF into cells, which can be called a “protein mediated electroporation”.

## 1. Introduction

The application of electricity in humans can be traced back to the 17th century when tissue damage was firstly observed, a phenomenon that is currently explained as an irreversible electroporation (EP) of the cellular membrane [1,2,3,4]. Despite the wide daily and clinical applications of electricity, it was not until 1982 when the first application of EP was designed to deliver exogenous material to the cytoplasm: using an electric field of 8 kV/cm by 5 μs, Neumann and cols. [5] reported the first transfection of genetic material guided by EP. Since the publication of this breakthrough, a significant understanding of the biophysics driving the EP process has accumulated [6,7]. To gain a modern understanding of EP, please refer to [8,9,10]. Notably, the definition of EP has remained intact for over 30 years: EP is the transient loss of semi-permeability of cell membranes subjected to electric pulses, leading to ion leakage, escape of metabolites, and increased cell-uptake of drugs, molecular probes, or DNA [6]. This technology is nowadays widely used for other applications than solely DNA transfection, such as electrochemotherapy [11,12], cold EP [13], tissue ablation [1,14], intracellular delivery [15], extraction of various compounds [16,17], and in the food industry [18,19,20,21]. More recently, a following step in the application of electric pulses was the development of High Intensity Nano Pulsed Electric Fields technology, known in the scientific community as nanosecond Pulsed Electric Field (nsPEF) or Nano Pulse Stimulation (NPS). This technology was developed in 1995 with the aim of improving the efficiency of an electric pulse, used in the industry to kill microorganisms responsible for biofouling in cooling systems that used untreated water from lakes, rivers, or the sea [22].

Since its first publication in 1995, the application of nsPEF has shown an explosive growth [23]. In general terms, the nsPEF technique consists of the delivery of a series of pulses of high electric fields (∼1–300 kV/cm) in the order of nanoseconds, even picoseconds, into biological tissues or cells. Their primary effect upon cells is the formation of membrane nanopores and the activation of ionic channels [24,25,26,27,28,29,30,31,32,33,34,35], having as a direct consequence an increment in the cytoplasmic concentration of Ca${}^{2+}$ [36,37,38,39], which triggers a set of signaling cascades, mainly ending in either apoptosis [40,41,42,43,44,45,46] or cell proliferation [47,48,49,50]. The unique characteristic that differentiates nsPEF from other types of electrostimulation is based on the timescale of nsPEF application, which lays in the order of the charging time of plasma membranes (∼100 ns in mammalian cells). This makes nsPEF capable of affecting inner organelles [36,51,52,53,54], making nsPEF a unique tool to manipulate and study cells. Notably, since each cell type has its own membrane charging time, nsPEF could be customized to be a highly cell-specific technology. This characteristic can be exploited by researchers in order to propose a wide spectrum of nsPEF applications, such as: activation of neurons [28,35,55,56,57,58] and myocites [59,60,61,62], wound healing [24,63,64,65], phenotype manipulation [50], modulation of gene expression [66,67,68,69,70,71], antiparasitic effects [72,73,74], enhancement of the immune response [75,76,77,78,79,80], cell proliferation [47,48,49,50], improvement in fermentation [81,82] and sterilization for the food industry [83,84,85], seed germination [86,87,88] and, most importantly, for the development of novel cancer therapies [23].

As one of the main effects of the application of nsPEF in cells is the change of permeabilization of cell membranes leading to the formation of membrane nanopores, nsPEF devices can also be defined as EP devices; indeed, the effect of nsPEF accomplishes with the EP definition: “a transient loss of semi-permeability of cell membranes subject to electric pulses, leading to ion leakage…” [6]. However, it is important to mention that despite general similarities between both, nsPEF may not be used for transfection because the induced nanopores are far from having a large-enough size to make membranes permeable to plasmids nor DNA fragments. Importantly, in spite of EP-induced cell membrane permeability changes, EP is not capable of producing the activation of ionic channels in the cell; one of the main effects of nsPEF.

From the biophysical point of view, the application of nsPEF induces, almost instantaneously, a voltage difference (ΔV) in cell membranes around 1.5 V [31]: up to three orders of magnitude above the cell resting potential (−80 mV). The electric field ($\vec{E}$) in the membrane given this ΔV ($\vec{E}=\Delta V/d$, where *d* is membrane thickness) generates a force over charged particles $\vec{F}=q\vec{E}$, with *q* being the net charge of each particle. Of note, this vectorial force acting over charged particles generates an electrochemical potential acting across the cellular membrane. In other words, the transmembrane (TM) potential elicited by the application of nsPEF is due to the fast rearrangement and accumulation of particles with opposite charges (the reaction potential) in each membrane interphase. For detailed theoretical considerations to better understand the effects of the application of nsPEF into cells, see Supplementary Information Section 1.

Despite the general consensus on the formation of nanopores by the application of nsPEF, an interesting controversy in the academic community arises regarding how voltage-gated channels (VGC) can be activated so quickly by nsPEF using electric stimuli in the order of nanoseconds, or even picoseconds. Thus far, the literature is conclusive: the gating mechanism in these channels occurs in the order of milliseconds [89,90]. Hence, additional data are needed to explain the nanosecond-scale gating occurring in VGC under the influence of nsPEF. Such data may generate new biophysical knowledge, leading to more sophisticated manipulations of nsPEF effects. To do so, Molecular Dynamics (MD) simulations emerge as a suitable option to explore, with atomistic resolution, both the kinetics and the thermodynamics of the nanosecond-scale gating occurring in VGC induced by the application of nsPEF. The application of MD simulations to study the effects of nsPEF opens an interesting field, which is currently scarcely explored. Previous works have simulated VGCs or their voltage-sensing domain (VSD) under the presence of an external electric field, causing TM potentials from 100 to 500 mV [91,92,93,94]. However, these simulations do not account for the intensity of an nsPEF that elicits TM potentials up to 1.5 V (which cannot increase due to the formation of nanopores), as concluded by both experimental and theoretical approaches [54,95,96,97,98,99]. In this work, we will focus on dissecting the effects of the application of nsPEF stimuli on the VSD of a voltage-gated (VG) Ca${}^{2+}$ channel. To do so, we will pay close attention to dissect the conformational changes occurring in both the membrane and the VSD, obtaining insights into both the mechanistic processes (kinetics) and the energy barriers (thermodynamics) describing the nanosecond-scale gating elicited by nsPEF.

**Figure 1:**
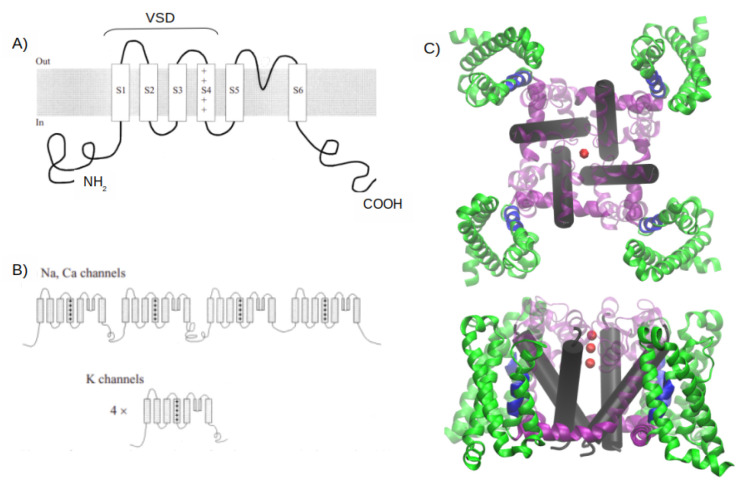
Structural overview of voltage-gated channels. (**A**) Transmembrane disposition of a single voltage-gated K${}^{+}$ channel subunit. There are six transmembrane regions, denoted by S1 through S6. (**B**) Structural organization of voltage-gated channels. Na${}^{+}$ and Ca${}^{2+}$ channels have four homologous repeats of the core motif in a single polypeptide chain; K${}^{+}$ channels are tetrameric assemblies of subunits with a single core motif. (**C**) A 3D representation of the crystallographic structure of an *Arcobacter butzleri* RM4018 VGCC in its open state (PDB ID: 4MS2). In green, the four VSD; in blue, the helical conformation 3${}_{10}$ in the S4 helices; in purple, the S4–S5 helices (not present in (**A**) or (**B**)) that connect the S5 and S6 helix; in light purple, the S5 helices and the rest of amino acids that connect to the S6 helix. In black, the cylinders of the S6 helices, and the red spheres represent three calcium atoms.

Na${}^{+}$, Ca${}^{2+}$ and K${}^{+}$ VGCs constitute a family of ubiquitous and structurally related integral membrane proteins [100] with therapeutical importance [101]. Voltage-activated Na${}^{+}$ and Ca${}^{2+}$ channels are composed of monomers with various auxiliary subunits, while voltage-activated K${}^{+}$ channels are tetramers. All of them present four repeated structures, formed by six α-helices, see Figure 1. Helices S1 to S4 form the VSD, and helices 5 and 6 form the pore domain [102]. The four VSD are ubiquitous to Na${}^{+}$, Ca${}^{2+}$ and K${}^{+}$ VGCs [92].

During VGC activation, the displacement of the charges tethered to the VSD gives rise to transient gating currents. Kinetic models indicate that during VGC activation, the VSD undergoes a complex conformational change encompassing several transitions [103,104,105]. Four main models have been proposed to rationalize the transfer of charge during VGC activation, all of them associated with the motion of the S4 helix [106,107]: the helical screw-sliding model [108,109], the kinetics model [110], the paddle model [111] introduced following the publication of the K${}^{+}$ channel (KvAP) structure [112], and the transport model [113]. Of note, in all these models, the S4 helix triggers the conformational change in VSDs in order to activate VGCs. This key role has been supported by experimental evidence indicating that charged amino acid residues present in the S4 helix constitute the channel gating charges [114,115]. This can be considered intuitive, given that the S4 helix has a net charge whose value is much higher than the other VSD helices. Each S4 helix has a different number of positively charged arginine residues interspersed by two neutral amino acids.

Apart from the key role of charged amino acid residues for the gating of VGCs, the composition of the cellular membrane also plays a crucial role, in particular, cholesterol acting in lipid rafts. Lipids rafts are defined as microdomains in cellular membranes having differential lipid composition [116,117]. In the original conceptualization of the lipid raft model, raft formation was based on preferential interactions between sphingolipids and cholesterol [118,119] and possibly other lipids such as gangliosides [120]. These data were obtained from cell membranes of epithelial cells [121,122], suggesting an equimolar mixture of cholesterol, saturated lipid-like sphingomyelin and unsaturated phosphatidylcholine, usually bearing two unsaturated acyl chains. Later, it became clear that this mixture cannot be extrapolated to all mammalian cell membranes. A constant in lipid raft composition is an increase of 3 to 5-fold the amount of cholesterol found in the surrounding bilayer [123].

Current literature proposes that the central role of lipid rafts organization in membrane domains is to sort, organize and maintain the structure and function of membrane proteins [124]. It is not yet clear whether lipid domains are recruiting freely diffusing proteins or if proteins could recruit a dynamic assembly of raft-forming lipids [125]. What is clear, however, is that the structure and function of membrane proteins depend on the membrane lipid composition where they are embedded [126,127]. An excellent review by Andersen et al. delves deeper into this topic [128]. The structure/function dependency of TM proteins with membrane composition is due to specific lipid–protein interactions and other general bilayer–protein interactions, which modulate the energetics and kinetics of protein conformational transitions. These interactions have a hydrophobic nature because they mainly arise from contacts between the membrane’s hydrophobic core and TM protein’s hydrophobic domains. A conformational change of a TM protein can perturb the protein/membrane interface, which could also alter the surrounding membrane, resulting in further membrane deformation. The energy cost for deforming the membrane is directly related to its composition, which in turn determines membrane properties such as bilayer thickness, intrinsic lipid curvature, elastic compression and bending modulus. Thus, membranes can be considered as an allosteric regulator of protein function [128,129].

To further explore the effects of nsPEF not only on the VSD of the VG Ca${}^{2+}$ channel but also on the cellular membrane, we performed MD simulations using two different configurations of the membrane; a pure 1-palmitoyl-2-oleoyl-sn-glycero-3-phosphocholine (POPC) bilayer—typically used in MD simulations—and a cholesterol/POPC bilayer, emulating a lipid raft environment. In both configurations, we evaluated the effects of an external electric field mimicking the application of an nsPEF. In doing so, we evaluated if the conformational changes occurring in the VSD may be related to the presence or absence of lipid rafts. Our results suggest that the activation of voltage-gated Ca${}^{2+}$ channels by a nsPEF may be due to major conformational changes elicited by the application of the external electric field. This work only contains data from molecular simulations. No actual experimental data are presented.

## 2. MD Simulations Methods

The GROMACS molecular dynamics package 2019.1 [130] was used to perform the simulations. In all simulations, the CHARMM36 force field was used to account for parameters describing bond length, angle bending, angle torsions and non-bonded interactions of the molecular systems [131]. Two rectangular simulation boxes were constructed, named Box 1 and Box 2. Box 1 contains 167 POPC molecules in a bilayer arrangement, 9811 H${}_{2}$O molecules, 1 Cl${}^{-}$ ion, in order to neutralize the system, and the VSD at the center of the bilayer. Box 2 contains 32 POPC and 96 cholesterol molecules (∼1:3 mol ratio) in a bilayer arrangement, 7709 H${}_{2}$O molecules, 1 Cl${}^{-}$ ion, in order to neutralize the system, and the VSD at the center of the bilayer. This high amount of cholesterol is not far from some biological membranes, such as the plasma membranes of red blood cells, and myelin membranes of Schwann cells isolating nerve axons with around 50 mol% of cholesterol [132]. Even more, the phase diagram of cholesterol and dimyristoyl-phosphatidylcholine (DMPC) shows a bilayer structure with 66 mol% of cholesterol [133]. Before the MD simulation, a steepest-descent minimization algorithm was applied to relax the molecular system, followed by a NVT/NPT equilibration of 100 ps for each thermodynamic ensemble. The equilibrated Box 1 has a size x/y/z of 7.65, 7.65 and 8.73 nm, respectively, and the equilibrated Box 2 has a size x/y/z of 5.84, 5.84 and 10.18 nm, respectively. These box sizes ensure that the VSD do not see its periodic images. The leap-frog integrator algorithm was used with an integration time step of 2 fs for integrating Newton’s equations of motion. Van der Waals interactions were simulated using the Lennard Jones potential (LJ), and long-range electrostatic interactions were calculated using the Particle Mesh Ewald (PME) method [134]. A cut-off of 1.2 nm was employed for the LJ and the electrostatic interactions. The linear constraint solver (LINCS) was used to restrain bond lengths [135]. Simulations were carried out in an NPT ensemble at 310 K and 1 bar, coupled to a Nosé–Hoover thermostat [136] and to a Parrinello–Rahman barostat, [137], with time constants of 1 and 5 ps for temperature and pressure, respectively. Simulations were performed with periodic boundary conditions in all directions. The TIP3P (transferable intermolecular potential with 3 points) water model [138] was employed in all simulations. TIP3P is an explicit 3-atom rigid water molecule with charges and Lennard–Jones parameters assigned to each of the 3 atoms. The program used to embed the VSD in the bilayer was the Membrane Builder module of CHARMM-GUI [139]. Each equilibrated box was simulated for 200 ns. After the 200 ns of simulation of Box 1, 20 replicas of 50 ns under an $\vec{E}$ = 0.1 V/nm, and 20 replicas of 50 ns under an $\vec{E}$ = 0.2 V/nm were performed. After 200 ns of simulation in Box 2, 20 replicas of 50 ns under a $\vec{E}$ = 0.1 V/nm, and 20 replicas of 50 ns under an $\vec{E}$ = 0.2 V/nm were performed. To increase the exploration of the conformational space, for Box 2 we executed 80 additional replicas of 50 ns under a $\vec{E}$ = 0.2 V/nm. The $\vec{E}$ direction was the same in all simulations, with the vectorial force oriented antiparallel to the z-axis and perpendicular to the bilayer. The VSD model was derived from the crio-electron microscopy at 3.60 Å of resolution, of a VGCC (PDB ID: 5gjv) isolated from *Oryctolagus cuniculus*.

## 3. Simulation Results

**Figure 2:**
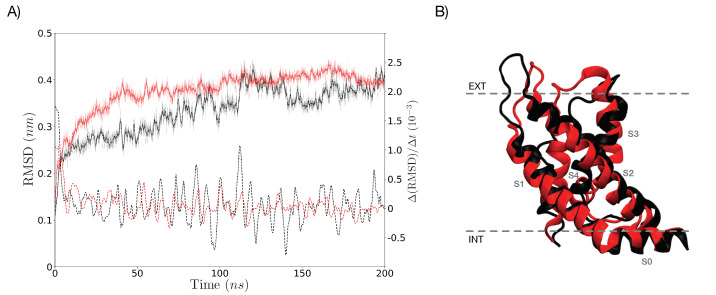
Comparison of both VSD structures after 200 ns of simulation without any external $\vec{E}$. (**A**) RMSD (after a lsq fit) as a function of time for the VSD in Box 1 (thin black line) and Box 2 (thin red line). RMSD values after a LOWESS smoothing appear in thick black and red lines. The derivative along time of the RMSD is shown using dashed lines. As seen, the RMSD derivative stabilizes very rapidly, fluctuating in the order of $10^{-3}$ nm, from 5 ns, up to the end of the simulation, denoting that the molecular systems have reached to equilibrium. (**B**) Overlapping with a RMSD = 0.4364 nm of the final conformations for both VSD structures in Box 1 (in black) and Box 2 (in red) after 200 ns of simulation without any external $\vec{E}$.

After 200 ns of simulation without any external $\vec{E}$, the VSD of both Box 1 and Box 2 conserved its native conformation, which is reflected by similar and stabilized root mean square deviation (RMSD) as a function of time. These results suggest that the incorporation of cholesterol in Box 2 did not have major consequences in the conformation of the VSD, see Figure 2. RMSDs calculations in all Figures were obtained using a comparison all-against-all, considering all atoms of the VSD model as a function of time. For a representation of Box 1 and Box 2 including the bilayer and the VSD final structure after 200 ns of simulation without any external $\vec{E}$, see Figure 5A.

**Figure 3:**
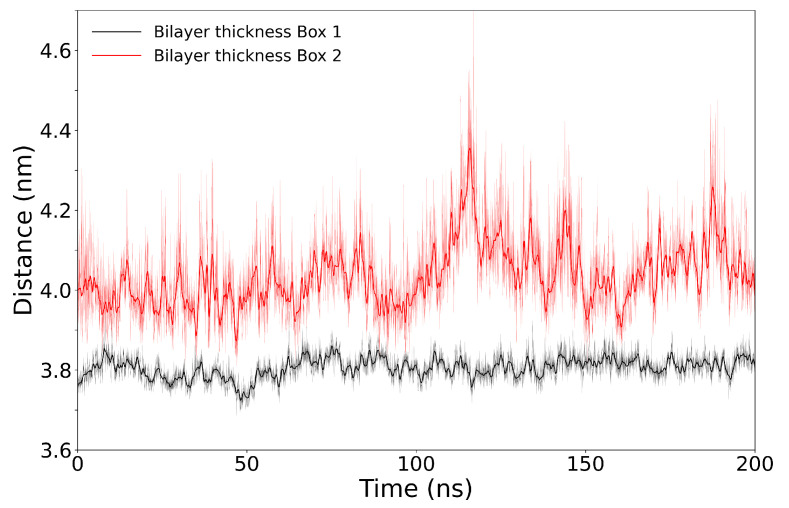
Thickness of the bilayer for Box 1 (thin black line) and Box 2 (thin red line) as a function of time, simulated for 200 ns. Represented by the average distance between the center of mass of the phosphate of each POPC molecule in one layer with respect to the complementary one. Values after a LOWESS smoothing appear in thick black and red lines, respectively

As mentioned before, the effective TM potential across the bilayer after applying an nsPEF using a continuous $\vec{E}$[140] depends on the thickness of the bilayer (d). The thickness of the bilayer was obtained by measuring the distance between the center of mass of the phosphate of each POPC molecule in one layer with respect to the complementary one; see Figure 3. For Box 1, the average thickness along the 200 ns simulation is 3.883 ± 0.26 nm (d${}_{Box1}$), while for Box 2, the average thickness resulted as 4.00 ± 0.85 nm (d${}_{Box2}$). The TM potential (ΔV) can be estimated using the following equation,

(1) $$\Delta V=\vec{E}d.$$

Thus, the TM potential resulting from a $\vec{E}$ = 0.1 V/nm and 0.2 V/nm in Box 1 is ∼0.38 ± 0.26 V and ∼0.77 ± 0.26 V, respectively. For Box 2, the resulting TM potential is 0.4 ± 0.85 and 0.8 ± 0.85 V, respectively. Importantly, resulting TM potentials from our simulations are below the limit (∼1.5 V) of the TM potentials induced by the application of nsPEF stimuli in cell membranes. Above this limit, the nsPEF-induced cell permeation hindered further increase of the TM potential [31]. It is important to mention that these calculations can also be done in the absence of ions (there is only one Cl${}^{-}$ in Box 1 and in Box 2) and they consider a homogeneous dielectric constant across the media.

### 3.1. Simulations under an External Electric Field of 0.1 V/nm

**Figure 4:**
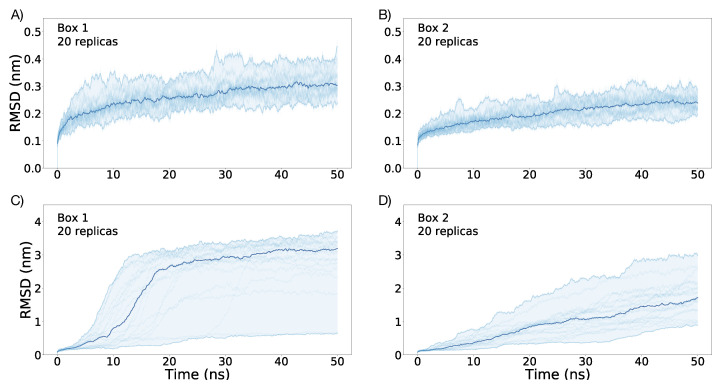
RMSD calculated using the *lsq fit* procedure in GROMACS as a function of time for all the replicas after 50 ns of simulation time under a $\vec{E}$ = 0.1 V/nm, (**A**,**B**); and under $\vec{E}$ = 0.2 V/nm, (**C**,**D**). RMSD trajectories obtained from each replica are plotted in light blue, and the largest deviation, the lowest deviation and the mean are depicted in dark blue, after the application of a LOWESS smoothing.

**Figure 5:**
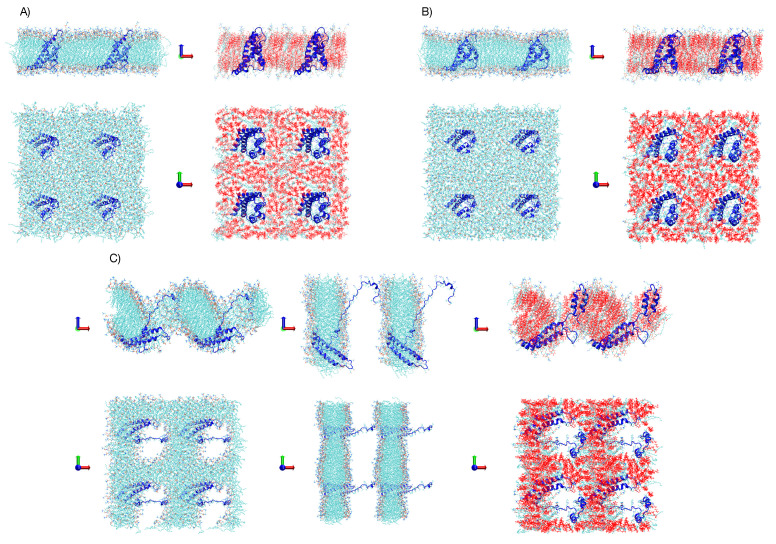
Bilayer and the VSD of Box 1 and 2 with 3 periodic images in x and y, views from beside and up. Cholesterol in Box 2 highlighted in red. (**A**) Box 1 and Box 2 after 200 ns of simulation under an $\vec{E}$ = 0.0 V/nm. (**B**) Box 1 and Box 2 after 50 ns of simulation under an $\vec{E}$ = 0.1 V/nm. (**C**) Box 1 and Box 2 after 50 ns of simulation under an $\vec{E}$ = 0.2 V/nm. On the left of the panel (**C**), an additional membrane/VSD representation at 15 ns of simulation is shown to illustrate that the disruption of the membrane in Box 1 starts from similar pores as observed in Box 2.

Using the final conformation of the 200 ns trajectory as a starting point, 20 replicas (simulation repetition with the same conditions) of each box were simulated under an $\vec{E}$ = 0.1 V/nm for 50 ns. As can be observed from Figure 4A,B, the VSD does not have major conformational changes in both boxes for all replicas. The smaller values of RMSD for Box 2 with respect to Box 1 for all replicas could be the result of different bilayer composition. The presence of cholesterol in Box 2 could render a more rigid bilayer, keeping the conformation of the VSD with fewer changes than that of the VSD from Box 5. Nevertheless, in both boxes, the external electric field does not seem to have major effects on the VSD conformation. For a representation of Box 1 and Box 2 including the bilayer and the VSD final structure after 50 ns of simulation using an $\vec{E}$ = 0.1 V/nm, see Figure 5B.

### 3.2. Simulations under an External Electric Field of 0.2 V/nm

An $\vec{E}$ = 0.2 V/nm was applied to the 20 replicas for additional 50 ns of simulation in both boxes. In the case of Box 1, for 18 of the 20 replicas, the VSD undergoes a fast conformational change, while the bilayer structure is drastically modified; the bilayer changes from being perpendicular to the z-axis to being parallel to the z-axis, see Figure 5C. For additional snapshots at intervals of 5 ns to illustrate this bilayer/VSD transition, see Supplementary Information Section 3 Figure S4, and for the RMSD of the bilayer showing this rearrangement, see Figure S5. The irreversible rearrangement of the bilayer prevents the analysis of any VSD conformational change that can be biologically meaningful. This was not the case in Box 2 since the amount of cholesterol made the membrane more resistant to an external electric field. Although the bilayer suffers an important rearrangement in the 20 replicas of Box 2, it preserves the bilayer structure, as can be seen in Figure 5C. Differences in the conformational change of the VSD between Box 1 and Box 2 under $\vec{E}$ = 0.2 V/nm can be followed by comparing their RMSD plotted in Figure 4C,D.

**Figure 6:**
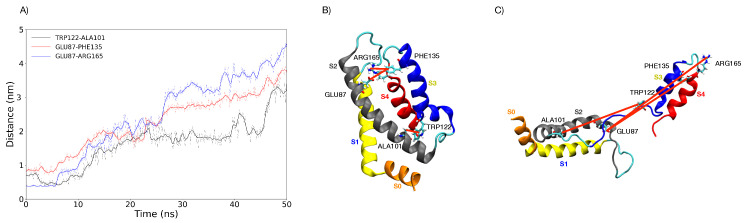
(**A**) Distances as a function of time between different VSD amino acid residues of a representative replica of Box 2 under an $\vec{E}$ = 0.2 V/nm during 50 ns. In black, the distance between TRP 122 and ALA 101; in red, the distance between GLU 87 and PHE 135; in blue, the distance between GLU 87 and ARG 165. (**B**) Representation of the amino acid residues plotted and their distance at the beginning of the simulation with $\vec{E}$ = 0.2 V/nm. (**C**) Representation of the amino acid residues plotted and their distance at the end of the 50 ns of a representative simulation under $\vec{E}$ = 0.2 V/nm. The S0–S4 helices are highlighted in orange, blue, gray, yellow and red, respectively.

The change in the bilayer/VSD structure follows similar paths for the 20 replicas in Box 2, characterized by two major rearrangements; pore formation and a partial unfolding with formation of two sub-domains containing two helices each. As a first approximation and to follow the conformational changes occurring in the VSD in Box 2 under an $\vec{E}$ = 0.2 V/nm, a set of distances between amino acid residues were obtained as a function of time. To do so, we identified a representative replica that was calculated considering the minimum distance between the RMSD of each final frame and the average RMSD computed from the ensemble of all final frames; see Figure 6. For additional snapshots at intervals of 5 ns of the VSD in the replica selected for Figure 6; see Supplementary Information Section 4, Figure S6.

To further analyze the conformational changes produced in the VSD, the positions of all charged amino acid residues in the VSD were identified. In Supplementary Information Section 5, Table S1 presents charged amino acid residues in the VSD, and in Figure S7, their position in the VSD is depicted. Interestingly, the VSD group movement to the x-y plane, including the S1 and S2 helices, may have a relation with the sub-domain charge distribution. To the external media, this group has a net charge of −2 e, and to the internal media, it has a net charge of +1 e. As the simulated external electric field generates force lines from +z to −z along the simulation box, perpendicular to the x-y plane, the force vector on this charged amino acid residue induces a torque that may play a relevant role in explaining this sub-domain torsion during the simulation.

**Figure 7:**
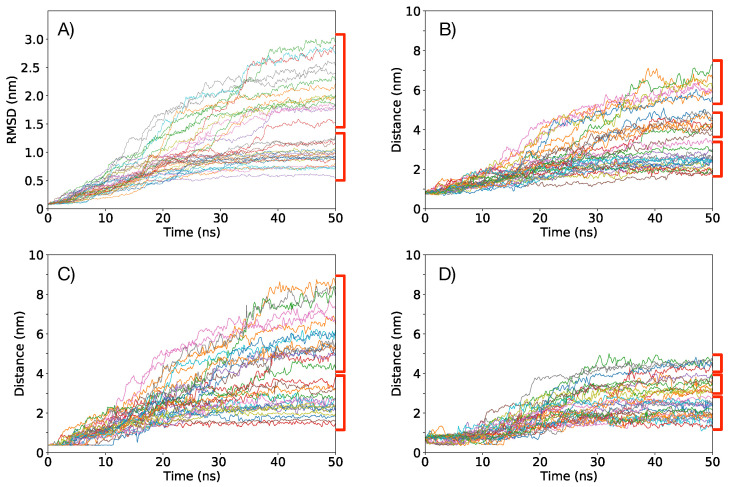
RMSD and measured distances of the VSD for the selected 34 replicas of Box 2 under a $\vec{E}$ = 0.2 V/nm for 50 ns. (**A**) RMSD as a function of time; (**B**) distance as a function of time between GLU 87 and PHE 135; (**C**) distance as a function of time between TRP 122 and ALA 101; (**D**) distance as a function of time between GLU 87 and ARG 165. Red brackets indicate groups of trajectories, suggesting possible clusterization of final VSD structures.

For a more comprehensive representation of the phase space describing the conformational changes on which the VSD structure may undergo under the influence of an $\vec{E}$ = 0.2 V/nm, 80 additional replicas of Box 2 were simulated under the same conditions, so to complete a total of 100 independent replicas. From these 100 replicas, 34 were selected for having their final conformation stable for more than 10 ns and considering other conditions further explained in Supplementary Information Section 6. Obtained values for RMSD and distances between amino acid residues of the VSD of the 34 replicas, suggesting a possible clusterization of the final structures, are depicted in Figure 7.

**Figure 8:**
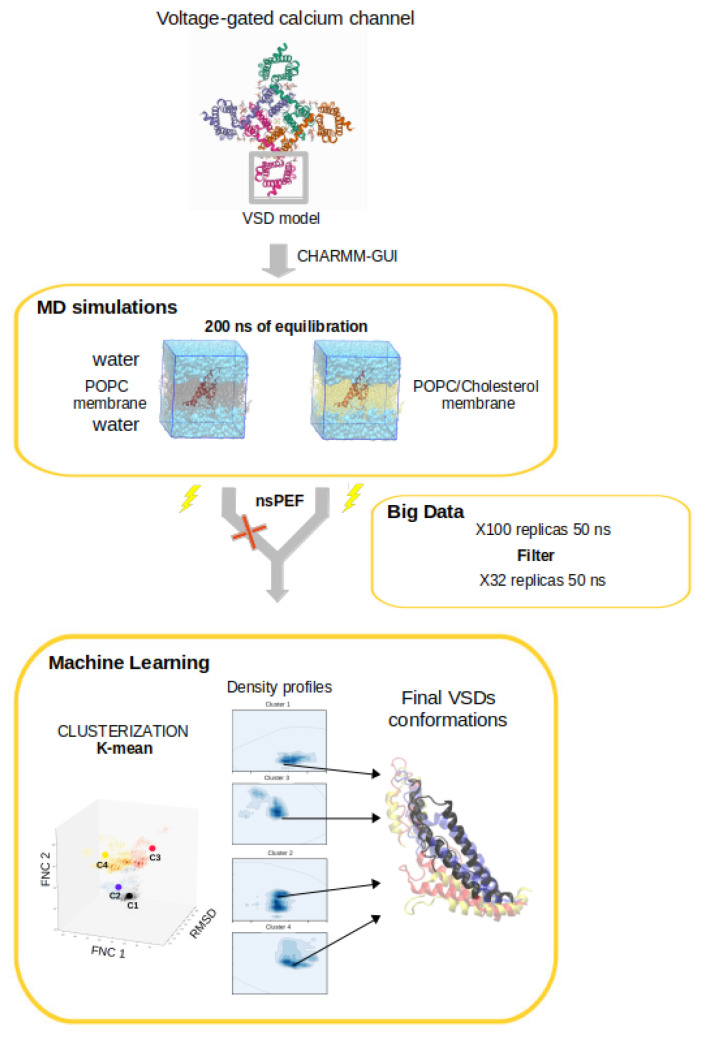
Flowchart of the study depicting the set of steps to determine the effect of the application of a set of external stimuli mimicking nsPEF. After the simulations, a 3D cube of data was produced considering the fraction of native contacts, and the RMSD, both calculated using as reference the initial frame. Once the 3D data cube was produced, an unsupervised K-means clustering was executed, leading to 4 main clusters denoted C1 to C4. From each cluster, the frame with the closest distance (by relying on the least squared method) to the center of mass of each cluster was selected as a representative structure.

For a better understanding of the steps needed to achieve the presented simulation results and the following ones, a flowchart is presented in Figure 8.

### 3.3. Simulations of Box 1 and Box 2 without VSD

As a control, we simulated Box 1 and Box 2 without the VSD following the same simulation protocol for the above simulations. These boxes were simulated for 200 ns and then five replicas under an external electric field of $\vec{E}$ = 0.1 V and 0.2 V/nm for 50 ns were performed for each box. The membrane RMSD of each replica indicates that the bilayer structure remains unperturbed under the action of the external electric field, $\vec{E}$ = 0.1 V/nm and 0.2 V/nm; see Supplementary Information Section 2, Figure S2. For a representation of the unperturbed bilayer structures, see Figure S3. These results indicate that the bilayer rearrangement due to an external electric field observed in Box 1 and Box 2 with the VSD is due to a cooperative interaction between the bilayer and the VSD.

### 3.4. Clusterization and Calculation of Free Energies

**Figure 9:**
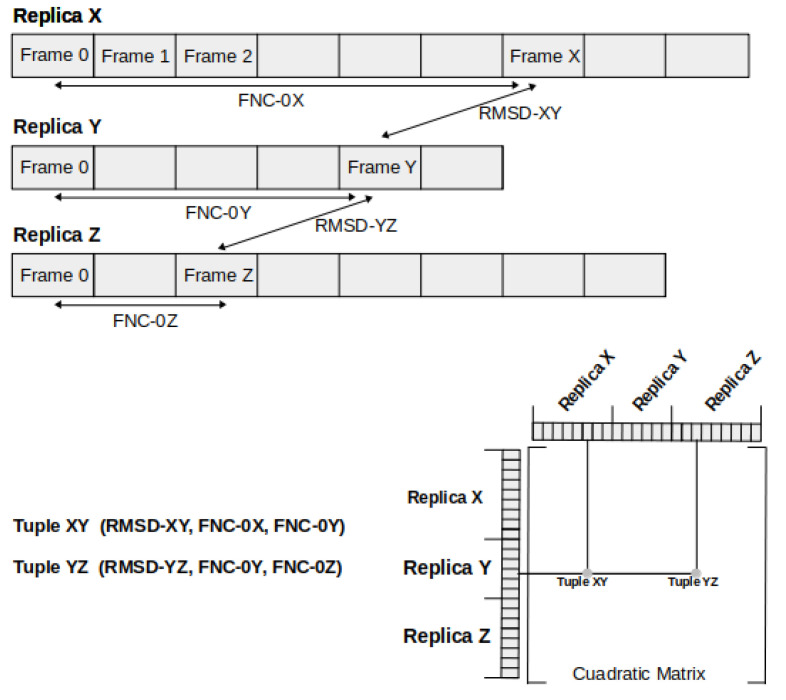
Schematic diagram exhibiting how the RMSD matrix and the set of tuples with FNC were constructed. RMSD-XY were obtained between Frame-X and Frame-Y, FNC-0X is the FNC between Frame-X and the initial Frame of the replica X, and FNC-0Y is the FNC between Frame-Y and the initial Frame of the replica Y. Thus, Tuple XY will be (RMSD-XY,FNC-0X,FNC-0Y). In the same manner, Tuple YZ was constructed. Obtaining a quadratic matrix with a total of $Tuples={\left(Total_{frames}\right)}^{2}$.

To determine which conformational changes occurring in the VSD are actual representatives among all the achieved conformations, considering the number of replicas and the performed simulations, we produced a clusterization by considering the fraction of native contacts (FNC) and the RMSD. The plot to afford the clusterization process was constructed considering tuples containing three coordinates, as follows. The first set of coordinates was obtained from an all-against-all computation of the RMSD between all the frames obtained from the 34 replicas, giving a total of 23,648,769 tuples. Each RMSD was associated with the FNC corresponding to the frame from which each RMSD tuple was calculated. In doing so, each tuple was defined as having one RMSD value and two FNC values. For further clarification on how the RMSD matrix and the set of tuples were constructed, please see Figure 9.

Clusterization of the 3D data cube composed of tuples containing the RMSD and the FNCs was performed using the unsupervised K-means method [141]. K-means is an unsupervised classification (clustering) algorithm that groups objects into *k* groups based on their characteristics. Grouping is done by minimizing the sum of distances between each object and the centroid of its group or cluster. The algorithm consists of three steps: (1) Initialization: once the number of groups, *k*, has been chosen, *k* centroids are established in the data space, for example, by choosing them randomly. (2) Assigning objects to centroids: each object in the data is assigned to its closest centroid. (3) Updating centroids: the position of the centroid of each group is updated, taking as a new centroid the position of the average of the objects belonging to said group. Steps 2 and 3 are repeated until the centroids do not move or move below a threshold distance at each step. To determine the number of clusters, the Elbow method was used [142]; see Supplementary Information Section 7. The Elbow method is a method that looks at the percentage of variance explained as a function of the number of clusters. This method exists upon the idea that one should choose a number of clusters so that adding another cluster does not give much better modeling of the data. The percentage of variance explained by the clusters is plotted against the number of clusters. The first clusters will add much information, but at some point, the marginal gain will drop dramatically and gives an angle in the graph. The correct *k* (number of clusters) is chosen at this point. The clusterization method yielded four clusters, named from 1 to 4 in descending order, with respect to their size. Cluster 1 contains 8,997,487 tuples, cluster 2 contains 5,592,895 tuples, cluster 3 contains 4,557,672 tuples, while cluster 4 contains 4,480,715 tuples. The cloud of the 23,648,769 tuples was plotted in a 3D graph, and each resulting cluster was colored differently (see Figure S9).

When considering the total number of executed independent replicas and the total number of produced frames, leading to a 3D data cube of more than 23 million points, we may argue that we have thoroughly sampled the phase space describing the conformational changes though which the VSD may undergo under the influence of the external stimuli mimicking nsPEF. Hence, the total set of frames represent, collectively, the kinetically accessible set of possible conformations of our molecular system. Therefore, we may assume that all possible conformations were sampled, reaching an equilibrium between clusters. Hence, we may compute the free energy between clusters by relying on,

(2) $$\Delta G^{0}=-RTln\left(\frac{P1}{P2}\right),$$

where *R* is the molar gas constant, *T* is the temperature and, in our case, the fraction $\frac{P1}{P2}$ represents the thermodynamical equilibrium between the number of tuples existing on each cluster. Calculated $\Delta G^{0}$ between all clusters appears in Table 1.

**Figure 10:**
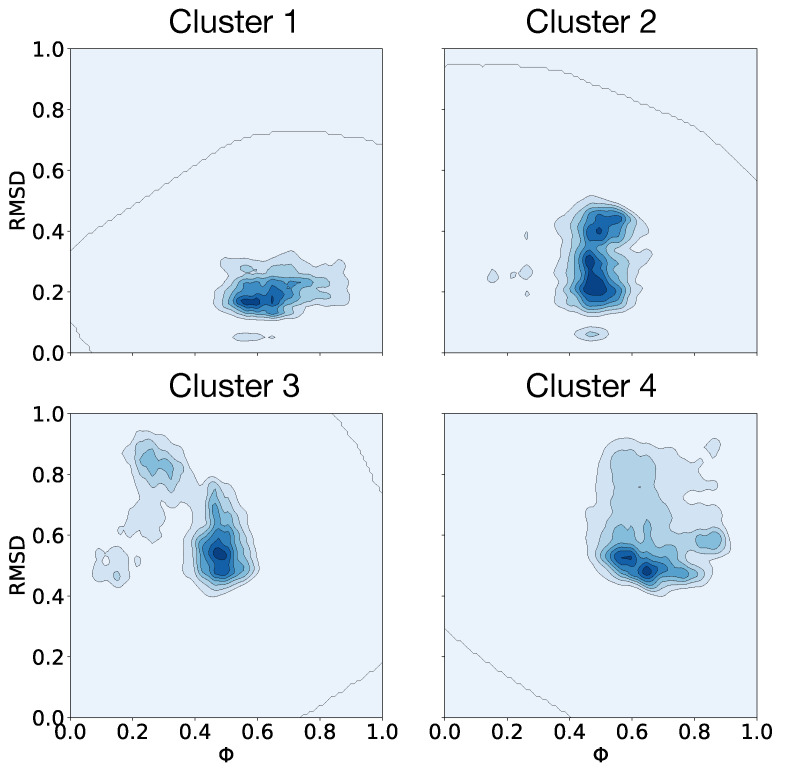
Density profiles for each cluster considering the RMSD and the FNC (Φ). Darker colors indicate denser regions.

A 2D representation depicting the density of the tuples for each cluster, considering the RMSD and the FNC, can be seen in Figure 10.

**Figure 11:**
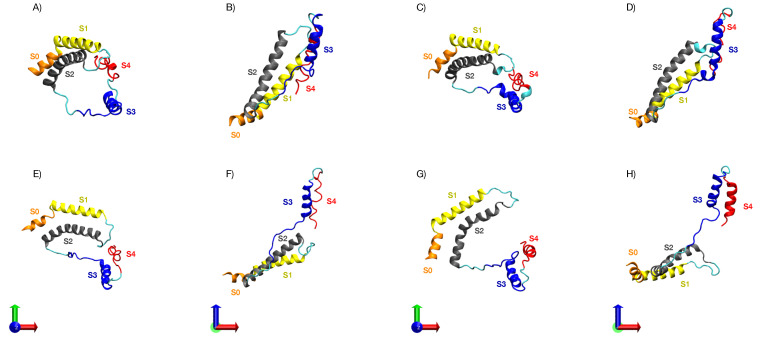
Cartoon representations of representative VSD conformations for each cluster from different perspectives. (**A**) Representative VSD of cluster 1, top view. (**B**) Representative VSD of cluster 1, side view. (**C**) Representative VSD of cluster 2, top view. (**D**) Representative VSD of cluster 2, side view. (**E**) Representative VSD of cluster 3, top view. (**F**) Representative VSD of cluster 3, side view. (**G**) Representative VSD of cluster 4, top view. (**H**) Representative VSD of cluster 4, side view. The S0 helix is highlighted in orange, the S1 helix is highlighted in blue, the S2 helix is highlighted in gray, the S3 helix is highlighted in yellow and S4 is highlighted in red.

From the density profile of each cluster, the closest tuple to the maximum density was obtained using the least squared method. This method was used over a kernel density estimation (KDE) constructed with all tuples using the quadratic error to find the tuple closest to the maximum density point in the KDE. Each of these tuples has an associated frame from the simulations that correspond to the most representative conformation for each cluster. In Figure 11, the conformations for each of these frames are represented in different perspectives. The frame numeration corresponds with their clusters.

### 3.5. Characterization of the Representative VSD for Each Cluster

A series of analyses performed on the initial frame and for each of the representative frames obtained per each cluster were performed, including, (1) radius of gyration (RGyr): a radial distance to a point that would have the same moment of inertia of the VSD actual distribution of mass; (2) solvent-accessible surface area (SASA): surface area of the VSD that is accessible to the solvent; (3) helix content; (4) distances between amino acid residues. These analyses are shown in Table 2.

## 4. Discussion

Without an external electric field, the VSD structure in both boxes resulted similar. This indicates that differences in membrane composition do not have major effects on the VSD conformation without an external electric field (Figure 2). Under the application of an external electric field of $\vec{E}$ = 0.1 V/nm, a differential evolution of the RMSD along time between both boxes becomes apparent: the average final RMSD of the first 20 replicas is 0.315 ± 0.047 nm and 0.243 ± 0.028 nm for Box 1 and Box 2, respectively (Figure 4A,B). This simulation result denotes the importance of membrane composition in the conformational change of the VSD under an external electric field. Changes on the physicochemical properties of membranes produced by the inclusion of cholesterol are well known; its inclusion mainly regulates membrane fluidity and permeability, as well as inducing the formation of coexisting phases and domains in the membrane [132]. The smaller average RMSD achieved by the VSD in Box 2 under an $\vec{E}$ = 0.1 V/nm suggests that cholesterol may be affecting the fluidity of the membrane, making it more rigid and retaining the VSD from undergoing conformational changes. This would explain in part the lesser conformational change of the VSD of Box 2 compared with that of the VSD in Box 1, as suggested when comparing the RMSD values. Considering the membranes as allosteric regulators of the structure and function of proteins becomes more evident when the changes in the VSD of Box 1 and Box 2, under an $\vec{E}$ = 0.2 V/nm, are compared. Both the VSD and membrane in Box 1, without cholesterol, suffer a major rearrangement that entails the loss of the bilayer structure (Figure 5C). This was not the case for Box 2, where cholesterol makes the membrane more resistant to the external electric field, allowing for the study of the conformational changes of the VSD (Figure 5C). The bilayer structure in Box 2 seems to be more resistant because it has fewer charged groups, making the membrane less susceptible to an external electric field; each POPC molecule has two opposite charges while cholesterol has none. RMSD values of the first 20 replicas of Box 2 under an $\vec{E}$ = 0.2 V/nm, as plotted in Figure 4D, suggests a possible clusterization of the final VSD conformation. However, this number of replicas was not enough to fully characterize the phase space in order to explore the free energy between the expected clusters, thus additional sampling was required. Therefore, 80 additional replicas were performed, albeit not all of them were able to reach an equilibrium in the 50 ns of simulation. In order to select the replicas, the main criterion we applied was that the VSD RMSD needed to be stable for at least 10 ns. Having the selected replicas, the phase space was explored satisfactorily, as could be observed in Figure S9. The K-means method for clusterization yielded four clusters. Given the size of the VSD and observing Figure 4D and Figure 7, four clusters (i.e., four different potential energy minima in conformational phase space) were considered as an expected value. What was not expected, but interesting nonetheless, were the small differences in free energy between clusters. All of them resulted below thermal noise (2.47 kJ/mol), which means that the VSD conformation under an $\vec{E}$ = 0.2 V/nm is oscillating in a thermal equilibrium between, at least, four different major conformations, represented by the conformations closest to the maximum density of the plots in Figure 10. From Figure 11, it can be observed that representative conformations of the VSD for clusters 1 and 2 are indeed very similar, with the same results for clusters 3 and 4. The major differences between clusters 1–2 and clusters 3–4 seem to be that the latter are formed by two sub-domains comprising two α-helices each, which are more separated than in the VSDs conformation for clusters 1 and 2. For a quantification of the differences between the representative VSD conformation for each cluster, a series of parameters were calculated, namely radius of gyration, solvent-accessible surface area, α-helix content and distances between amino acid residues, see Table 2. This data corresponds with the qualitative observations obtained from Figure 11. Clusters 1–2 and clusters 3–4 have a similar radius of gyration and solvent-accessible surface area between them. The α-helix content is more difficult to interpret, but with respect to the initial conformation, it is clear that the VSD conformation for each cluster loses an important α-helix content, with the least probable conformation (cluster 4) being the one that has the lowest α-helix content. This indicates that major conformational changes preserve more α-helix content. Comparing the radius of gyration with respect to the initial conformation (Table 2), all VSDs conformations for each cluster present higher values, reflecting an unfolding process occurring in all VSD, up to a larger extent for clusters 3 and 4 than for clusters 1 and 2. This is consistent with the qualitative observation of Figure 11. Considering the solvent-accessible surface area, all values are higher than that of the initial conformation, suggesting that the change in the VSD conformation involves a displacement of the VSD from the bilayer to the solvent. A relevant phenomenon (not fully characterized in detail in this work and will be further explored elsewhere) is the pore formation occurring in the VSD of Box 2 under an $\vec{E}$ = 0.2 V/nm (see Figure 5C). The maintenance of the integrity of the POPC/Chol membrane without the VSD under an $\vec{E}$ = 0.2 V/nm (see Figure S3F) suggests that the incorporation of VSD into this system induces a cooperative structural rearrangement in both the bilayer and the VSD of Box 2. Of note, this process leads to a pore formation inside the VSD, initiated by a pore-like defect in which the four-helical bundles of the VSD unfold into two smaller two-helical sub-domains.

## 5. Conclusions

Overall, this work was focused on providing insights on the mechanism of activation of a VG Ca${}^{2+}$ channel under the application of an external field applied in the order of nanoseconds, mimicking an nsPEF. As was discussed in the introduction, the mechanism of activation in such short timescales must be different from the physiological one described in the literature. Hence, two main conclusions can be obtained from this work. First, membrane composition, specifically cholesterol content, is fundamental for the response of the VSD to an external electric field, confirming that the membrane is an allosteric regulator, preserving both the structure and function of membrane proteins. Second, the extent of the conformational changes occurring in the VSD conformation under an $\vec{E}$ = 0.2 V/nm suggests that the activation of a VG Ca${}^{2+}$ channel by a nsPEF may be due to major VSD rearrangements compared with the physiological conformational changes described in the literature. Moreover, achieving a ΔG between representative clusters of the final conformations of VSD that lies below thermal noise confirms that at least during the simulated timescales, the conformational changes occurring in the VSD are thermodynamically reversible. This knowledge could provide important hints towards the understating of the biophysical laws governing the nanosecond-scale gating elicited by nsPEF. Last but not least, the conformational rearrangements of the VSD leading to a pore formation under an $\vec{E}$ = 0.2 V/nm in which two smaller two-helical sub-domains come apart can be the starting point to propose a novel effect of the application of nsPEF into cells: the formation of nanopores through a phenomenon we termed “protein-mediated electroporation”.

## Figures and Tables

**Table 1:** Free energy values between clusters.

Clusters	$\Delta G^{0}$ (J/mol)
1–2	−1225.5
1–3	−1753.0
1–4	−1796.9
2–3	−527.6
2–4	−571.5
3–4	−43.9

**Table 2:** Radius of gyration (RGyr), solvent-accessible surface area (SASA), helix content and distances between amino acid residues of the VSD of the initial frame and of each representative frame of each cluster. Distance-1: distance between GLU 87 and PHE 135. Distance-2: distance between TRP 122 and ALA 101. Distance-3: distance between GLU 87 and ARG 165.

Frames	RGyr (nm)	SASA (Å${}^{2}$)	Helix Content (%)	Distance-1 (Å)	Distance-2 (Å)	Distance-3 (Å)
Initial frame	1.605	9106.8	76.0	7.4	7.6	3.9
Frame 1	2.007	10,642.7	60.3	27.3	27.2	21.3
Frame 2	2.181	10,480.4	67.8	27.9	28.3	32.7
Frame 3	2.795	11,745.8	65.3	40.9	48.7	46.5
Frame 4	2.601	11,806.6	55.4	32.9	41.2	50.0

## Data Availability

All scripts and data can be found in https://github.com/DLab/article_nspef_membranes accessed on 26 June 2021.

## Supplementary Materials

The following are available online at https://www.mdpi.com/article/10.3390/membranes11070473/s1.

## Abbreviations

The following abbreviations are used in this manuscript:

nsPEF	Nanosecond Pulsed Electric Field
NPS	Nano Pulse Stimulation
VSD	Voltage Sensing Domain
VGCC	Voltage-Gated Calcium Channel
VGC	Voltage-gated ion channels
$\vec{E}$	Electric Field
TM	Transmembrane
POPC	1-palmitoyl-2-oleoyl-sn-glycero-3-phosphocholine
FNC	Fraction of Native Contacts
SASA	Solvent-Accessible Surface Area
ΔG	Free Energy
