# Supplementary material for: Exploring the Conformational Changes Induced by Nanosecond Pulsed Electric Fields on the Voltage Sensing Domain of a Ca2+ Channel
Source: Membranes (Basel). 2021 Jun 26;11(7):473. doi: 10.3390/membranes11070473 (PMC8303878; doi:10.3390/membranes11070473)
Supplement: Supplementary file 1 [file membranes-11-00473-s001.zip › Supporting_Information_corrected_Round_3.pdf]

Supplementary Information

Exploring the conformational changes induced by Nanosecond Pulsed Electric Fields on the Voltage Sensing Domain of a Ca^{2+} Channel

A.R. Ruiz-Fernández *[1,2]‡, L. Campos[1,2]‡, F. Villanelo[1,2], Sebastian E. Gutiérrez-Maldonado [1,2], and T. Perez-Acle*[1,2,3]

[1]*Computational Biology Lab, Fundación Ciencia & Vida, Santiago, Chile*
[2]*Facultad de Ingeniería y Tecnología, Universidad San Sebastian, Santiago, Chile*
[3]*Centro Interdisciplinario de Neurociencia, Universidad de Valvaraíso, Valvaraíso, Chile*
‡ These authors contributed equally to this work.

***Corresponding author**: aruiz@dlab.cl; tomas@dlab.cl

1 Theoretical considerations on nsPEF: time and intensity, the key elements

It is important to emphasize that Nanosecond Pulsed Electric Field (nsPEF) is a novel technology relying on electromagnetic radiation, but considering a completely different way than that of standard ionization radiation. The application of an electric field in a cell does not imply the absorption of energy by any molecule, as commonly occurs with the application of ionizing radiation. The fundamental effect of nsPEF in living systems is the movement of charged species. It is a completely different phenomena that can be situated between electrostatics and electrodynamics, basically because the nano time scale of the pulse creates an electric field that is changing in time over membranes. An excellent review thoroughly addresses this point [1]. The underlying theory explaining if the application of nsPEF is actually affecting inner or outer membranes is scrambled, mainly because authors in the field have different theoretical approaches, and thus there are no converging conclusions. The same happens

when different works try to reduce the cell to an electric circuit, all of them with their own approach. However, most authors agree that the keystone dictating the location of nsPEF effect is the time elapsed between the nsPEF initiation and the finalization of membrane charging. It is a common missunderstanding, for first time readers in the field, to assume that the $\vec{E}$ delivered by a nsPEF would instantaneously rise the membrane $\vec{E}$. In the same way than in a RC circuit, the condenser (i.e., plasma membranes) does not charge fully until a certain time lapse, which is related with the RC time constant ($\tau = RC$, where R is the resistance and C the capacitor's capacitance). The equation that describes the capacitor's voltage increase over time in a RC circuit is:

$$V(t) = V_0 e^{-t/\tau}, \tag{1}$$

where V_0 is the capacitor's voltage at time $t = 0$ and $V(t)$ is the capacitor's voltage at a certain time t. Importantly, $V(t)$ has an exponential growth, which results in the capacitor reaching 68% of its charge capacity at $t = \tau$, and almost maxing out its charge at around 4τ. Similarly, the rise in membrane voltage as a function of time, and the dependency of the time constant membrane (τ_m) with the surrounding media can be defined as [2]:

$$V_g(t) = 1.5aE_0 cos\theta(1 - e^{-t/\tau_m}), \tag{2}$$

$$\tau_m = aC_m(1/2\sigma_e + 1/\sigma_i), \tag{3}$$

where $V_g(t)$ is the voltage generated across the plasma membrane of a spherical cell, a is the cell radius, θ is the polar angle measured with respect to the electric field E_0, τ_m is the relaxation time constant [3] which is also known as membrane charge time, charging time constant [4–8] or charging time [4, 9, 10], C_m is the membrane capacitance per unit of area, and σ_e and σ_i are the external and internal conductivity, respectively. It is important to address that the cell radius also have relevancy in electroporation [11]. Equation 2 describes an exponential growth for $V_g(t)$ with a limit of $1.5aE_0 cos\theta$. For mammalian cells, τ_m is estimated to be around $\sim 100ns$ [7, 12].

It is important to clarify that τ_m does not actually represent the membrane charge time, instead it represents the time when the membrane reaches 63% of its charge capacity, further reaching 95% in 3τ [6]. Thus, τ_m could be underestimated in some studies. Taking this into account, we will follow the writing using the same language as current field-articles.

Getting back to the controversy raised in the former section (regarding inner or outer membranes), a theoretical approach explains how nsPEFs affect the interior of cells while common electroporation does not. This can be easily understood with an electrostatic model of the cell, considering it as a solid-metal-conducting sphere. When an electric field is applied to a conducting sphere, its electrons migrate to the anode which, after a characteristic time, results in an asymmetric charge distribution creating a self-induced electric field around the sphere, completely nullifying the external one and resulting in a zero-electric field inside the sphere. In cells occurs basically the same, only in this case the moving charges are ions instead of electrons, causing charge distribution equilibrium to be reached in much longer times, as seen in Figure S1. As the characteristic time to nullify the external electric field in cells is in the order of microseconds, during electroporation, where pulses last longer than microseconds, the internal electric field in cells becomes zero, thus the cell's interior does not present any major perturbations. This is not the case when the pulse duration is under the microsecond scale as occurs in nsPEF, where the internal electric field is not able to nullify the external one, thus explaining with this model why the internal membranes are affected with nsPEFs.

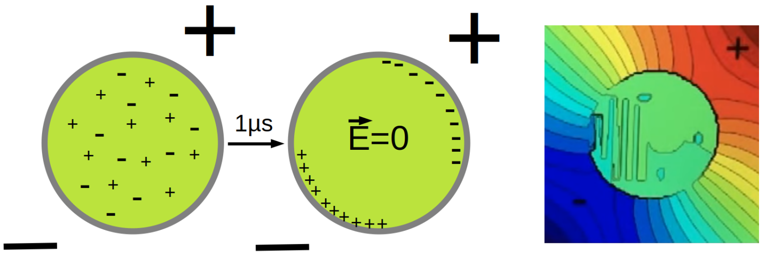

Figure S1: Schematic representation of charges movement inside the cell under the application of an external electric field, and how, once the equilibrium is reached, the electric field inside the cell is nullified

2 Simulation Controls, Boxes without VSDs

To determine if a possible bilayer rearrangement may commands the rearrangement of the VSD structure under the external electric field, simulations of Box 1 and Box 2 without the VSD following the same simulation protocol were performed. To do so, Box 1 and Box 2 without the VSD were simulated for 200 ns and then 5 replicas under an external electric field of $\vec{E}$=0.1 V/nm and 0.2 V/nm for 50 ns were performed for each Box. The membrane RMSD of each replica suggests that the bilayer structure remains unperturbed under the action of the external electric field, $\vec{E}$=0.1 V/nm and 0.2 V/nm, see Figure S2. For a representation of the unperturbed bilayer structures, see Figure S3.

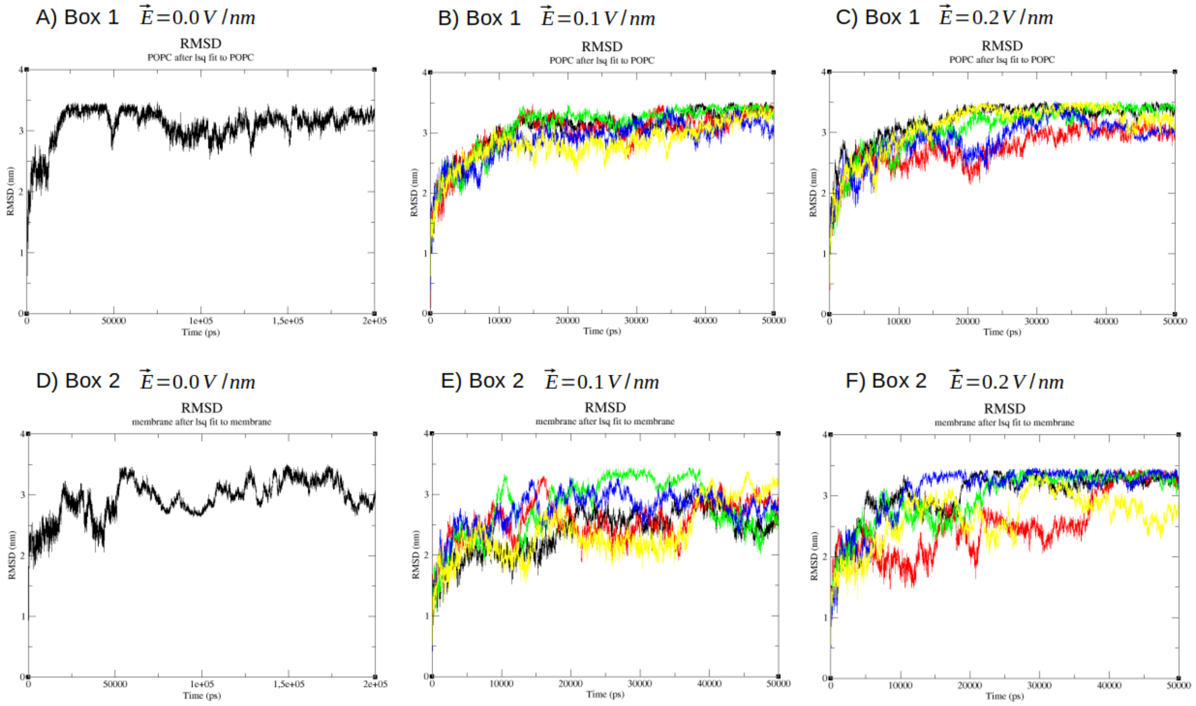

Figure S2: Bilayer RMSDs of Box 1 and Box 2 without the VSD. A) Bilayer RMSD as a function of time of Box 1 without an external electric field. B) Bilayer RMSD as a function of time of five replicas of Box 1 under an $\vec{E}$=0.1 V/nm. C) Bilayer RMSD as a function of time of five replicas of Box 1 under an $\vec{E}$=0.2 V/nm. D) Bilayer RMSD as a function of time of Box 2 without an external electric field. E) Bilayer RMSD as a function of time of five replicas of Box 2 under an $\vec{E}$=0.1 V/nm. F) Bilayer RMSD as a function of time of five replicas of Box 2 under an $\vec{E}$=0.2 V/nm.

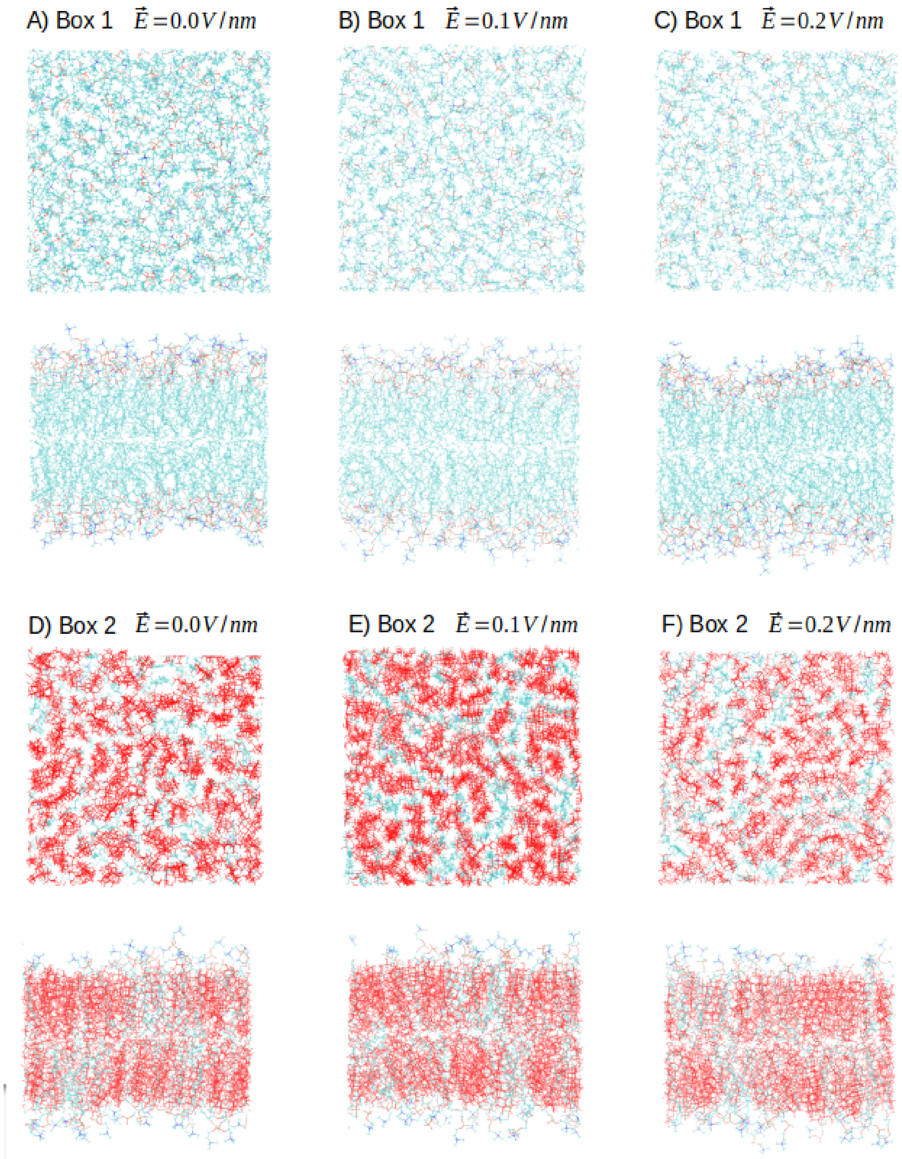

Figure S3: Bilayer representation of Box 1 and Box 2 without the VSD. Cholesterol is highlighted in red. A) Bilayer representation of Box 1 without an external electric field. B) Bilayer representation of a representative replica of Box 1 under an $\vec{E}$=0.1 V/nm. C) Bilayer representation of a representative replica of Box 1 under an $\vec{E}$=0.2 V/nm D) Bilayer representation of Box 2 without external electric field E) Bilayer representation of a representative replica of Box 2 under an $\vec{E}$=0.1 V/nm F) Bilayer representation of a representative replica of Box 2 under an $\vec{E}$=0.2 V/nm.

3 Analysis of bilayer rearrangement of Box 1 under an $\vec{E}$=0.2 V/nm

Bilayer in Box 1 under an $\vec{E}$=0.2 V/nm undergoes a mayor rearrangement, in which the plane of the bilayer changes from being perpendicular to the z-axis to being parallel to the z-axis. Figure S4 shows snapshots with the membrane and the VSD obtained at intervals of 5 ns. The RMSD of the membrane (POPCs) shows that the transition occurs mainly between 10 ns and 30 ns after the stimulus of the external electric field of $\vec{E}$=0.2 V/nm, see Figure S5.

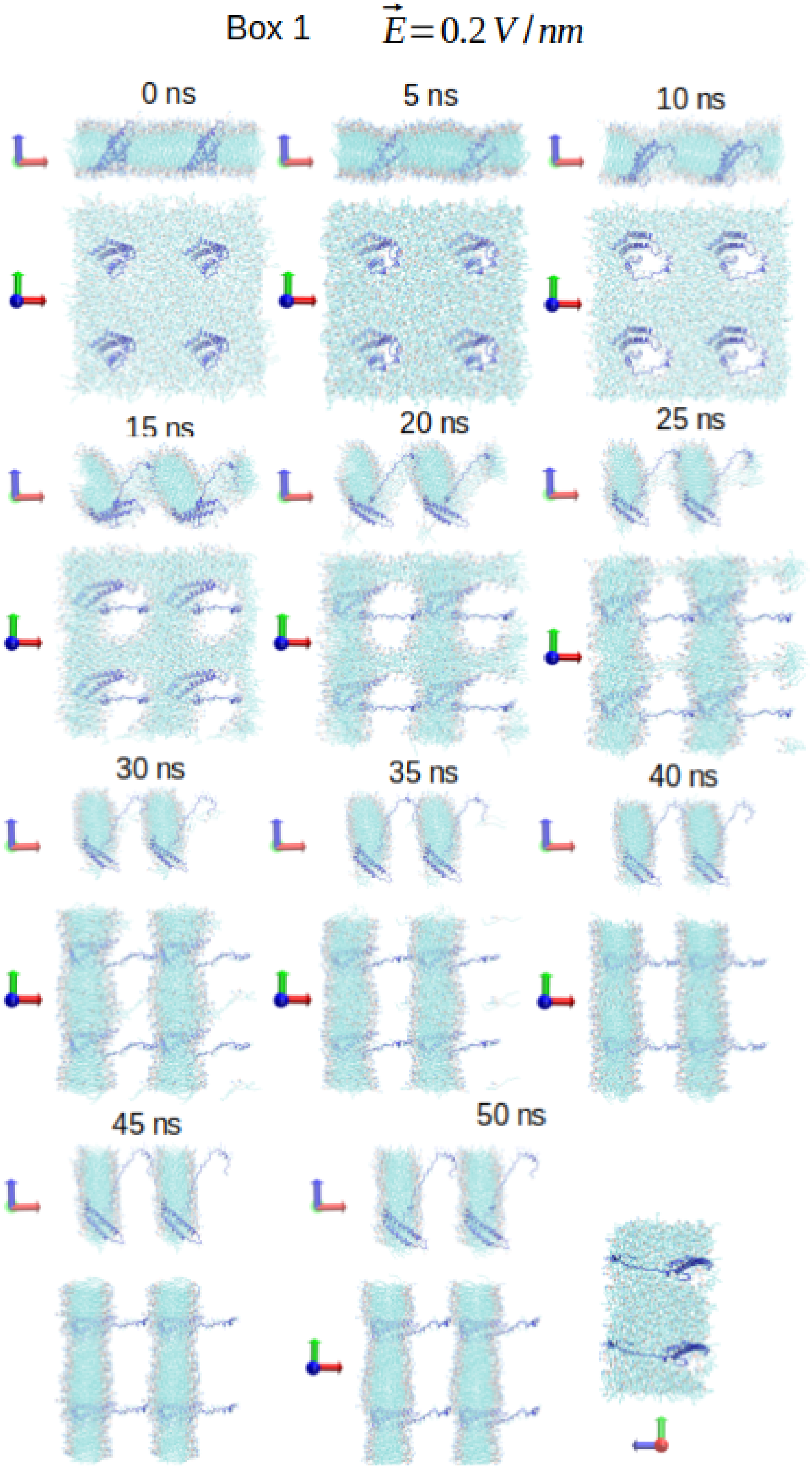

Figure S4: Snapshots with the membrane and the VSD obtained at intervals of 5 ns of one replica of Box 2 under an $\vec{E}$=0.2 V/nm for 50 ns. Each frame shows the bilayer/VSD perpendicular to the Y-axis and perpendicular to the Z-axis. The frame at 50 ns shows additionally a frame perpendicular to the X-axis.

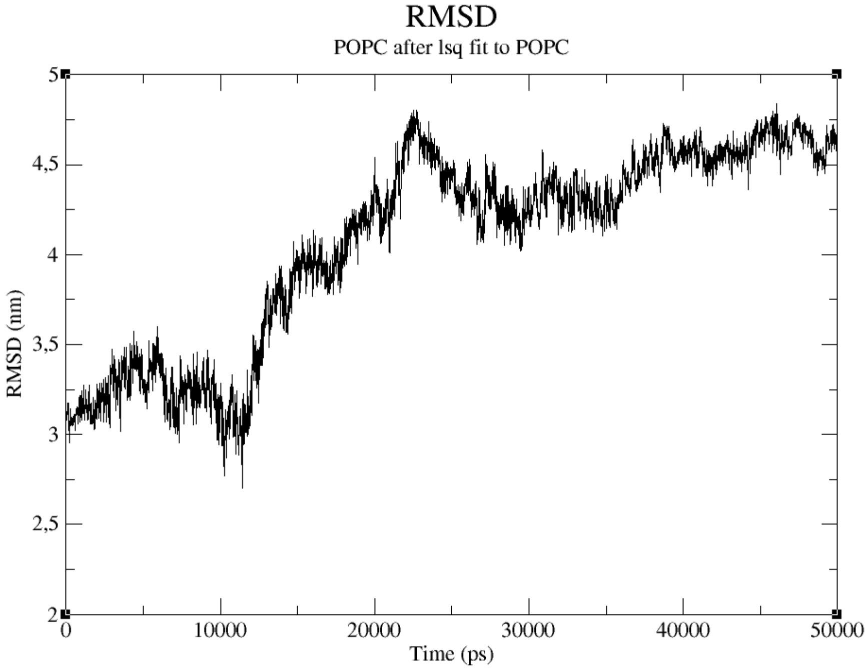

Figure S5: RMSD of the membrane of the same replica analyzed in Figure S4; Box 2 under an $\vec{E}$=0.2 V/nm for 50 ns.

4 Additional snapshots to follow VSD conformational change of Box 2 under an $\vec{E}$=0.2 V/nm

To visually follow the change in conformation of the VSD in Box 2 under an $\vec{E}$=0.2 V/nm snapshots of VSD at intervals of 5 ns were obtained, see Figure S6. The replica from which the snapshots were obtained is the same one used for Figure 6 in the main text. From these snapshots it can be observed that between 30 ns and 35 ns, helices S1 and S2 suffer a major torsion ending both helices in the x-y plane. S3 and S4 seem to maintain their orientation with respect to the arbitrary axis. It can also be observed that the structure of the S4 helix fluctuates between an α-helix secondary structure and the absence of it, maybe due to the high charge density associated with the four positively-charged arginines in its sequence that makes the helix more susceptible to external electric fields.

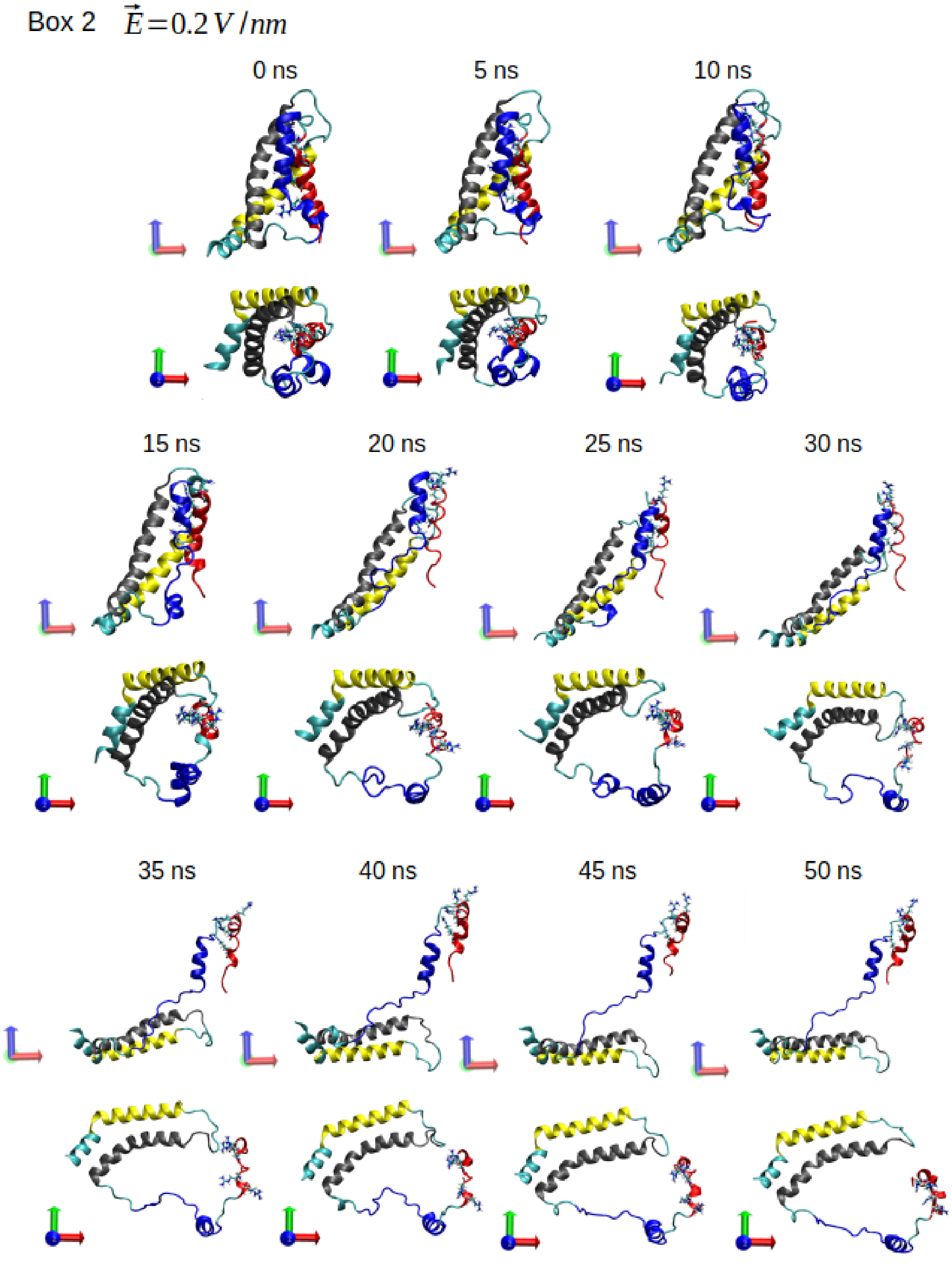

Figure S6: Snapshots of VSD at intervals of 5 ns of a representative replica of Box 2 under an $\vec{E}$=0.2 V/nm. Each snapshot shows the VSD perpendicular to the Y-axis and perpendicular to the Z-axis. The S1 helix is highlighted in yellow, the S2 helix is highlighted in grey, the S3 helix is highlighted in blue and the S4 helix is highlighted in red. The arginines of S4 helix are represented in licorice.

5 VSD charged amino acid residues

Table S1 shows the number of charged amino acid residues per helix and in the rest of the protein (loops and terminal helix). It is important to address that although the VSD present histidines in CHARMM36 Force Field, they are neutral in the structural context of the VSD. In Figure S7 the charged amino acid residues in the VSD are presented.

Charged amino acids residues	S1-helix	S2-helix	S3-helix	S4-helix	Rest
Arginine +1	–	–	1	4	1
Lysine +1	1	2	–	–	2
Glutamate -1	2	3	–	–	1
Aspartate -1	–	–	2	–	2
Total charge	+1	-1	-1	+4	0

Table S1: Number of charged amino acid residues for each helix and for the rest of the VSD.

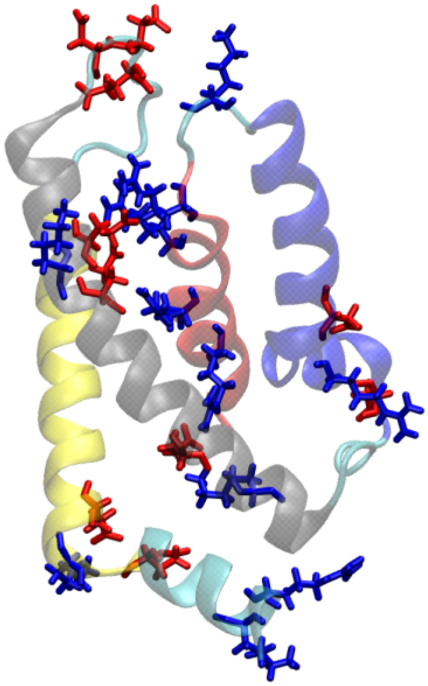

Figure S7: Representation in licorice of the charged amino acid residues in the VSD. In blue the cationic amino acid residues, in red the anionic amino acid residues. The VSD represented in cartoon with the S1 helix is highlighted in transparent yellow, the S2 helix is highlighted in transparent grey, the S3 helix is highlighted in transparent blue, and the S4 helix is highlighted in transparent red.

6 Selection of replicas for Clusterization and Free Energies

Two criteria were used to select the replicas. The first is related to the achievement of a near-to-equilibrium condition. To do so, the first derivative of the RMSD had to be close to zero for at least 10 ns, in order to have some certainty that the selected replicas have arrived to a stable conformation. The second criteria is to extrapolate the derivative average as an additional data to establish if the replica arrived to a stable conformation or not. The derivative was obtained by deriving the RMSD fitted using with the lowess regression method. The increment in the derivative in the last nanoseconds was due to a bias in the fitting of the extremes of the RMSD with the lowess method, and is not representative of any RMSD change. See Figure S8 to view the RMSD derivative and the cutting line in each replica selected.

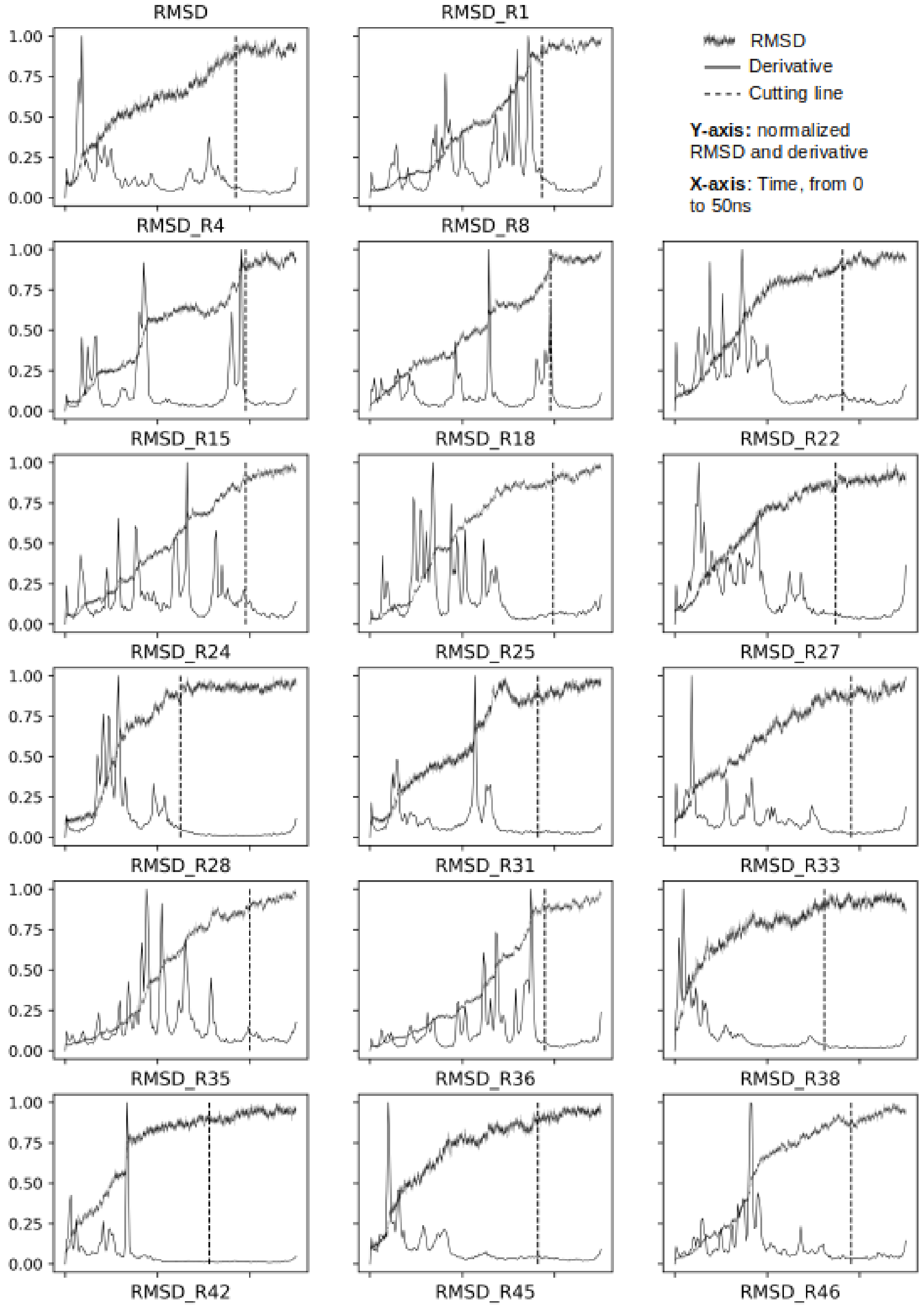
RMSD
RMSD_R1
RMSD
Derivative
Cutting line
Y-axis: normalized RMSD and derivative
X-axis: Time, from 0 to 50ns
RMSD_R4
RMSD_R8
RMSD_R15
RMSD_R18
RMSD_R22
RMSD_R24
RMSD_R25
RMSD_R27
RMSD_R28
RMSD_R31
RMSD_R33
RMSD_R35
RMSD_R36
RMSD_R38
RMSD_R42
RMSD_R45
RMSD_R46
1.00
0.75
0.50
0.25
0.00

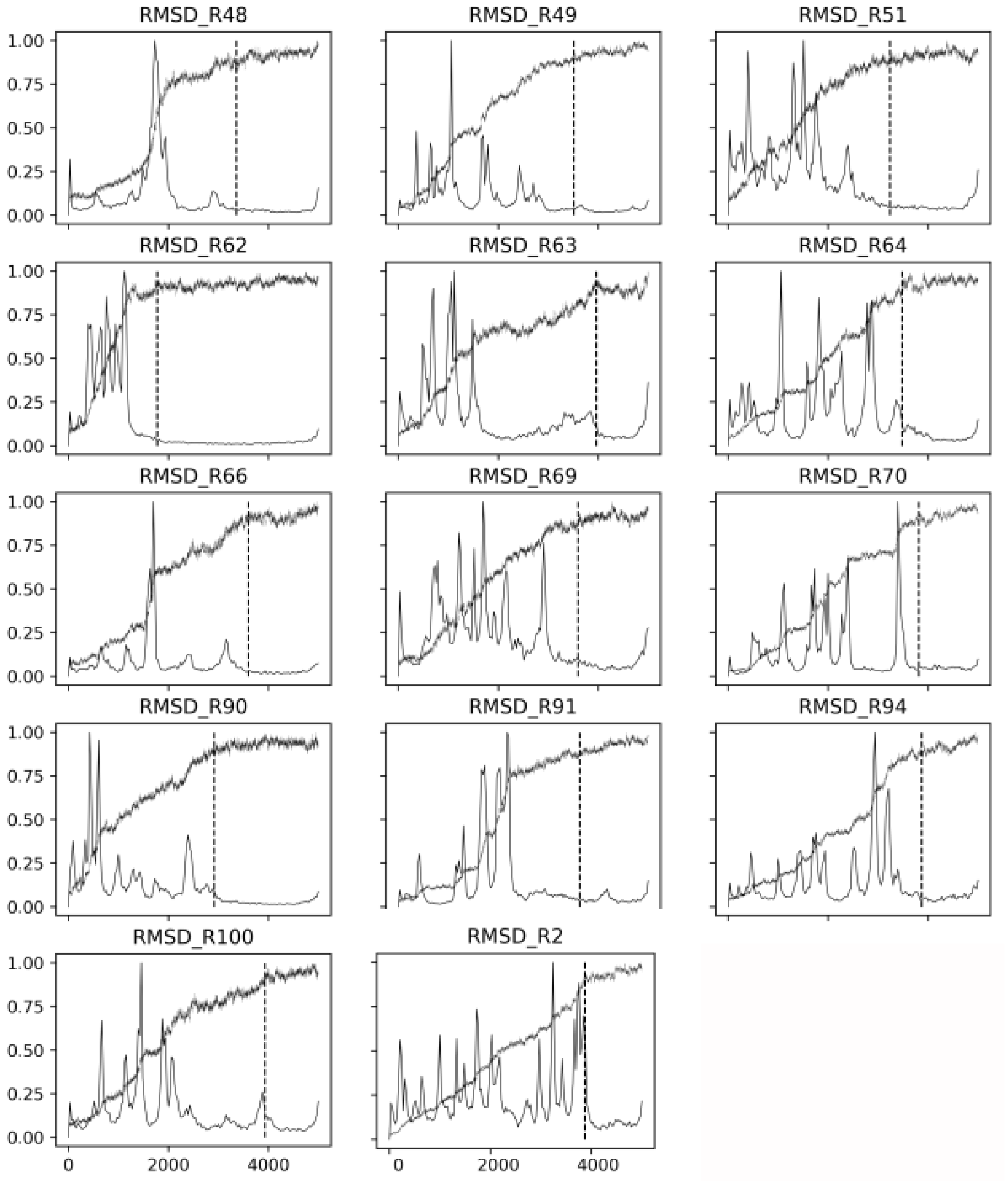

Figure S8: Cutting line from of the replicas analyzed.

The cloud of the 23,648,769 tuples was plotted in a 3D graph, and each resulting cluster was differentially coloured, see Figure S9.

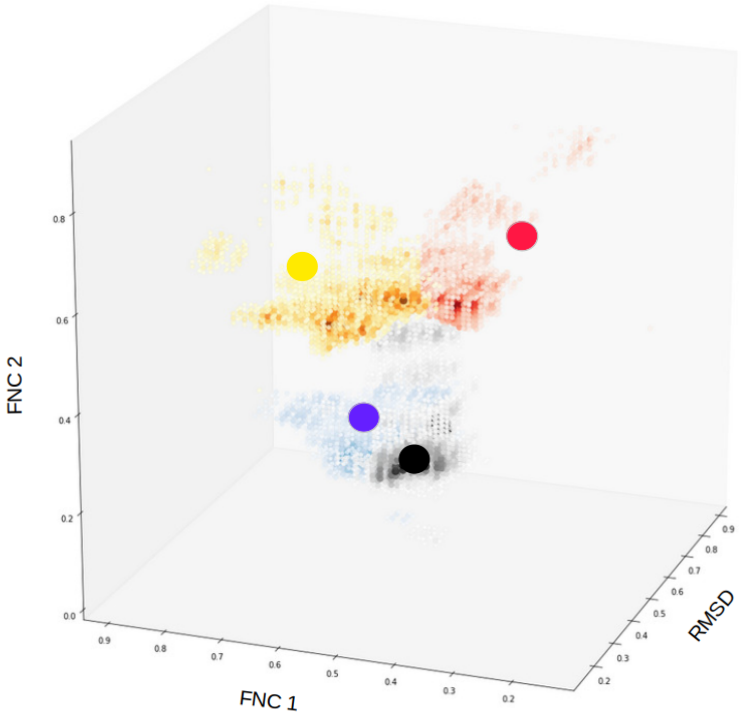

Figure S9: 3D graph of the resulting clusters formed by clouds of tuples. Cluster 1 in black, cluster 2 in blue, cluster 3 in red and cluster 4 in yellow. Normalized values.

7 Elbow method

-The best score after applying the Elbow method using inertia to the total of tuples was four clusters, see Figure S10

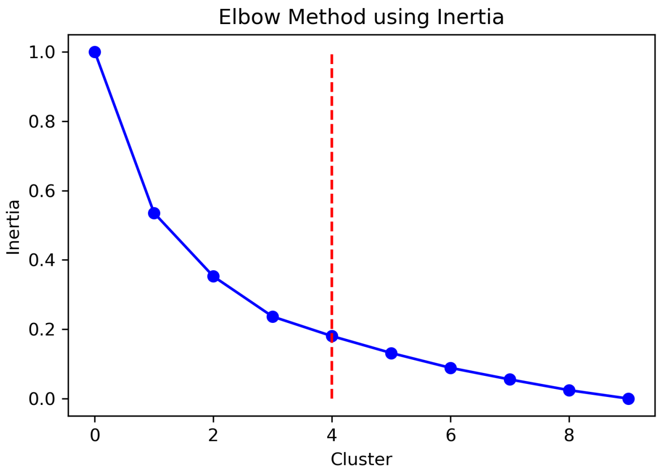

Figure S10: Cutting line from of the replicas analyzed. Normalized RMSD.

References

[1] Qin Hu, RP Joshi, and KH Schoenbach. Simulations of nanopore formation and phosphatidylserine externalization in lipid membranes subjected to a high-intensity, ultrashort electric pulse. *Physical Review E*, 72(3):031902, 2005.

[2] Ulrich Zimmermann and Garry A Neil. *Electromanipulation of cells*. CRC press, 1996.

[3] KJ Müller, VL Sukhorukov, and U Zimmermann. Reversible electropermeabilization of mammalian cells by high-intensity, ultra-short pulses of submicrosecond duration. *The Journal of membrane biology*, 184(2):161–170, 2001.

[4] Karl H Schoenbach, Stephen J Beebe, and E Stephen Buescher. Intracellular effect of ultrashort electrical pulses. *Bioelectromagnetics: Journal of the Bioelectromagnetics Society, The Society for Physical Regulation in Biology and Medicine, The European Bioelectromagnetics Association*, 22(6):440–448, 2001.

[5] Karl H Schoenbach, Amr Abou-Ghazala, Terry Vithoulkas, Ravmond W Alden, R Turner, and Stephen Beebe. The effect of pulsed electrical fields on biological cells. In *Digest of Technical Papers. 11th IEEE*

International Pulsed Power Conference (Cat. No. 97CH36127), volume 1, pages 73–78. IEEE, 1997.

[6] Juergen F Kolb, Susumu Kono, and Karl H Schoenbach. Nanosecond pulsed electric field generators for the study of subcellular effects. *Bioelectromagnetics*, 27(3):172–187, 2006.

[7] P Thomas Vernier, Yinghua Sun, Laura Marcu, Cheryl M Craft, and Martin A Gundersen. Nanoelectropulse-induced phosphatidylserine translocation. *Biophysical journal*, 86(6):4040–4048, 2004.

[8] Karl H Schoenbach, Ravindra P Joshi, Stephen J Beebe, and Carl E Baum. A scaling law for membrane permeabilization with nanopulses. *IEEE Transactions on Dielectrics and Electrical Insulation*, 16(5):1224–1235, 2009.

[9] Thiruvallur R Gowrishankar, Axel T Esser, Zlatko Vasilkoski, Kyle C Smith, and James C Weaver. Microdosimetry for conventional and supra-electroporation in cells with organelles. *Biochemical and biophysical research communications*, 341(4):1266–1276, 2006.

[10] Tina Batista Napotnik, Matej Reberšek, P Thomas Vernier, Barbara Mali, and Damijan Miklavčič. Effects of high voltage nanosecond electric pulses on eukaryotic cells (in vitro): A systematic review. *Bioelectrochemistry*, 110:1–12, 2016.

[11] Aparna Agarwal, Imants Zudans, Emily A Weber, Jessica Olofsson, Owe Orwar, and Stephen G Weber. Effect of cell size and shape on single-cell electroporation. *Analytical chemistry*, 79(10):3589–3596, 2007.

[12] P Thomas Vernier, Yinghua Sun, Laura Marcu, Sarah Salemi, Cheryl M Craft, and Martin A Gundersen. Calcium bursts induced by nanosecond electric pulses. *Biochemical and biophysical research communications*, 310(2):286–295, 2003.
