# Supplementary figures and images for: Exploring the Conformational Changes Induced by Nanosecond Pulsed Electric Fields on the Voltage Sensing Domain of a Ca2+ Channel
Source: Membranes (Basel). 2021 Jun 26;11(7):473. doi: 10.3390/membranes11070473 (PMC8303878; doi:10.3390/membranes11070473)

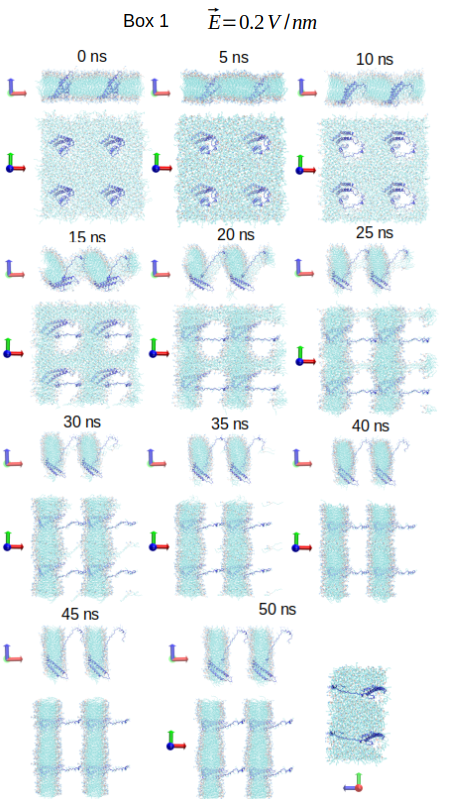

Supplement: Supplementary file 1 [file membranes-11-00473-s001.zip › Additional_frames.png]

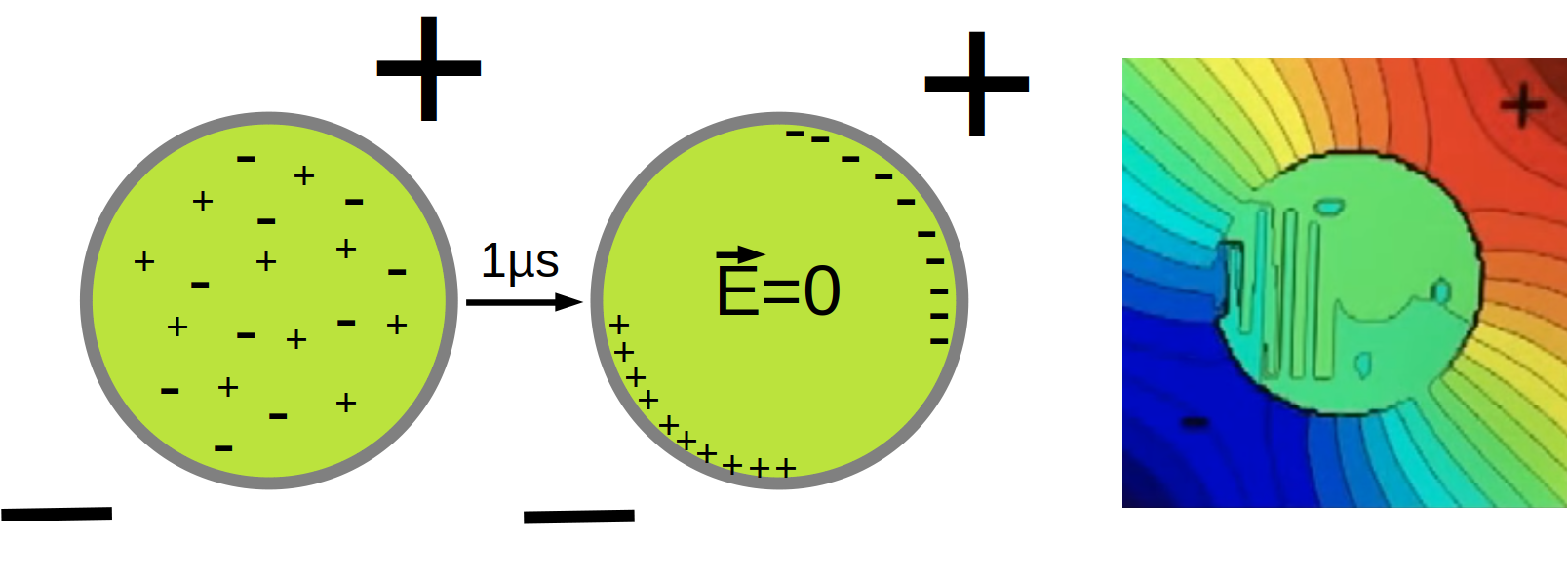

Supplement: Supplementary file 1 [file membranes-11-00473-s001.zip › celula_E_2.png]

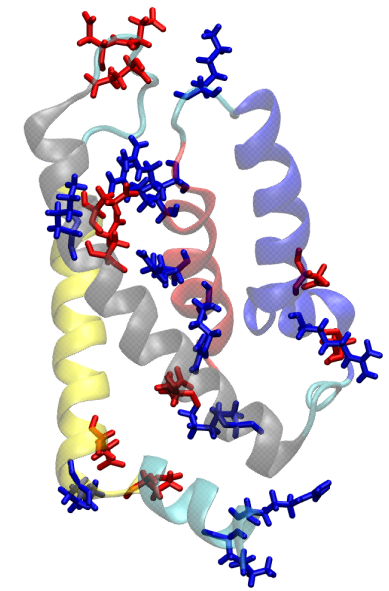

Supplement: Supplementary file 1 [file membranes-11-00473-s001.zip › charges_vsd.png]

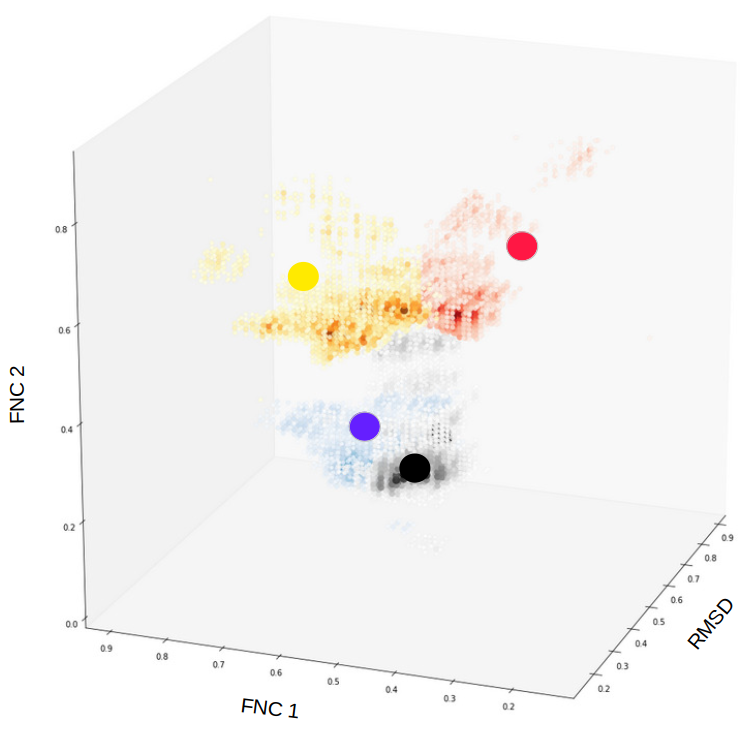

Supplement: Supplementary file 1 [file membranes-11-00473-s001.zip › cluster2.png]

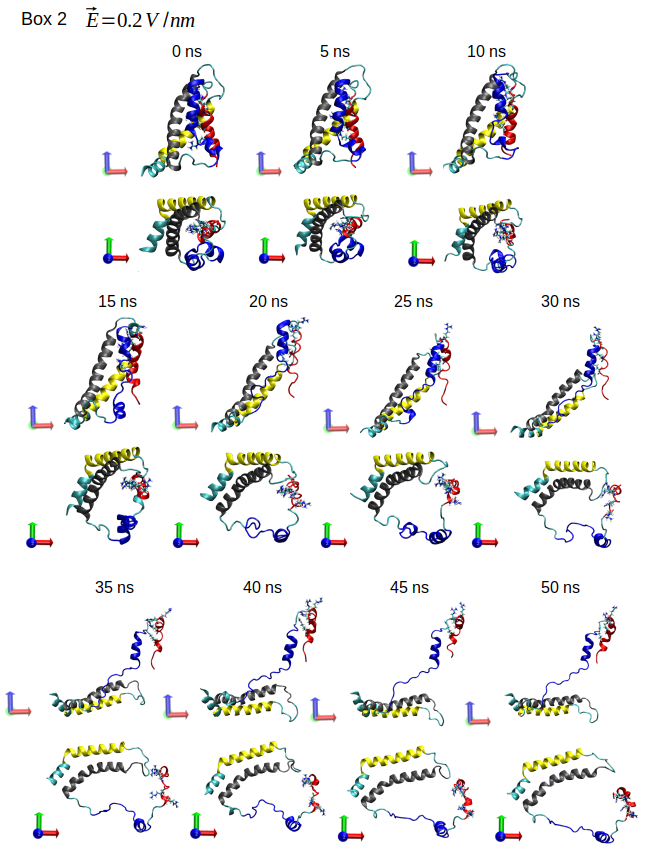

Supplement: Supplementary file 1 [file membranes-11-00473-s001.zip › conformation_correction.png]

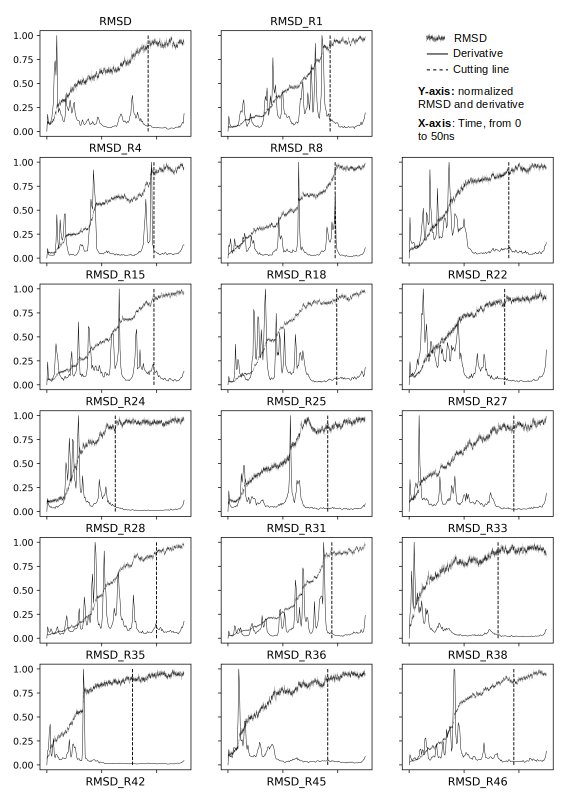

Supplement: Supplementary file 1 [file membranes-11-00473-s001.zip › derivadas_1.png]

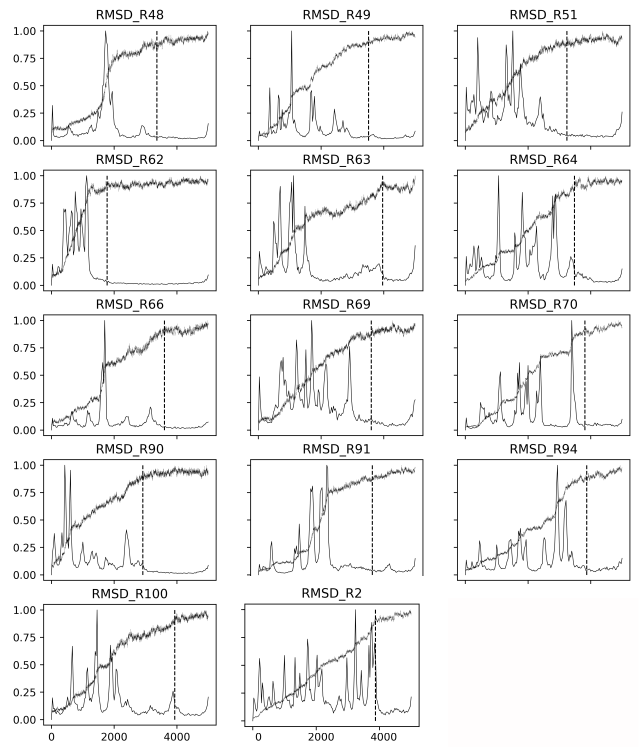

Supplement: Supplementary file 1 [file membranes-11-00473-s001.zip › derivadas_2.png]

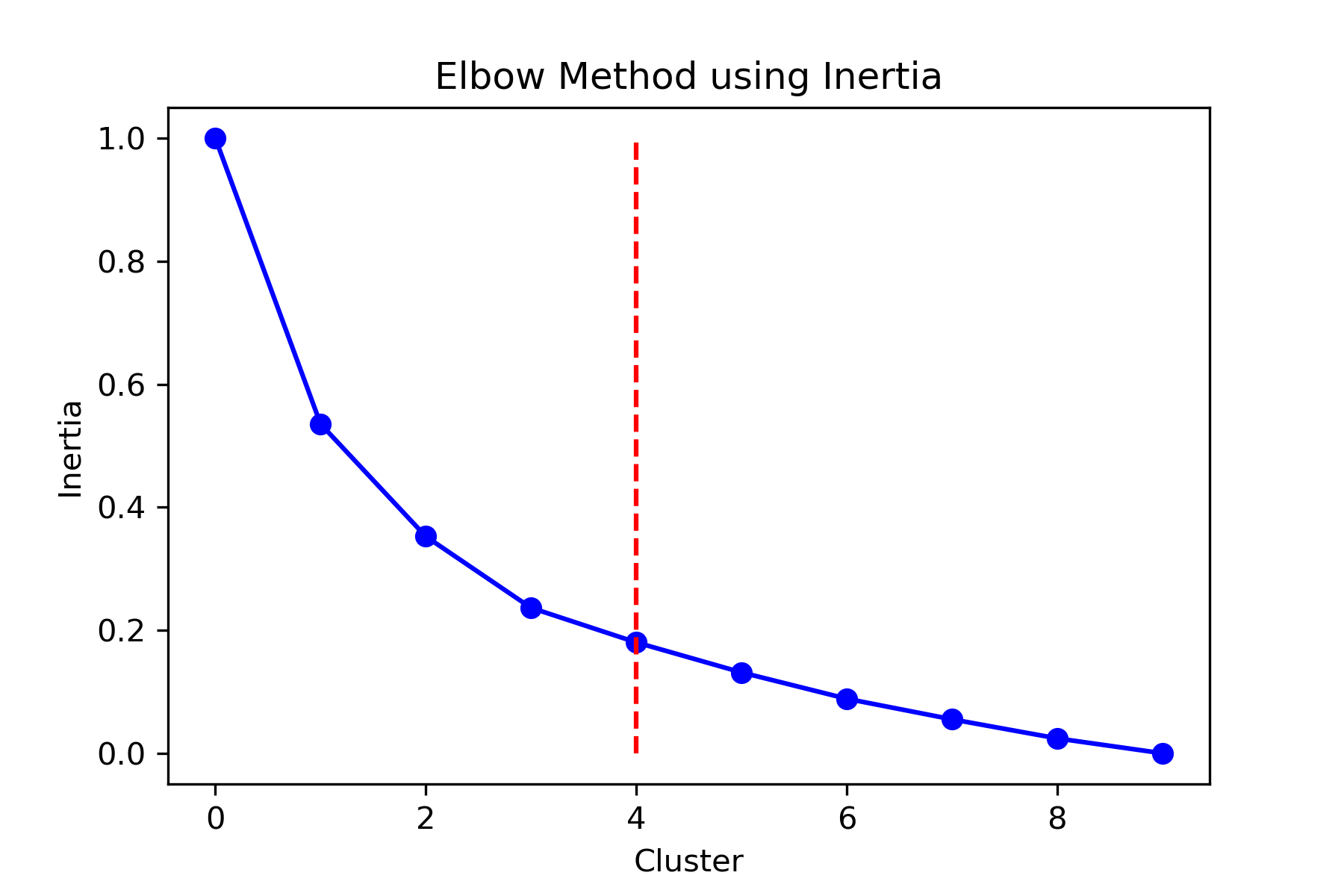

Supplement: Supplementary file 1 [file membranes-11-00473-s001.zip › Elbow.png]

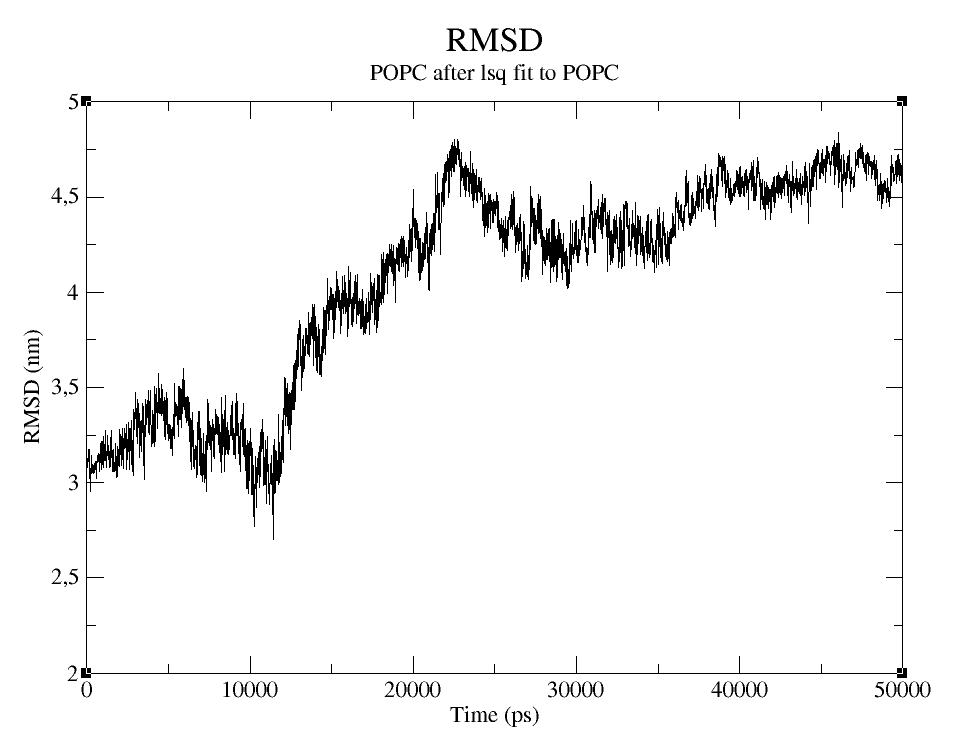

Supplement: Supplementary file 1 [file membranes-11-00473-s001.zip › rmsd_frames.png]

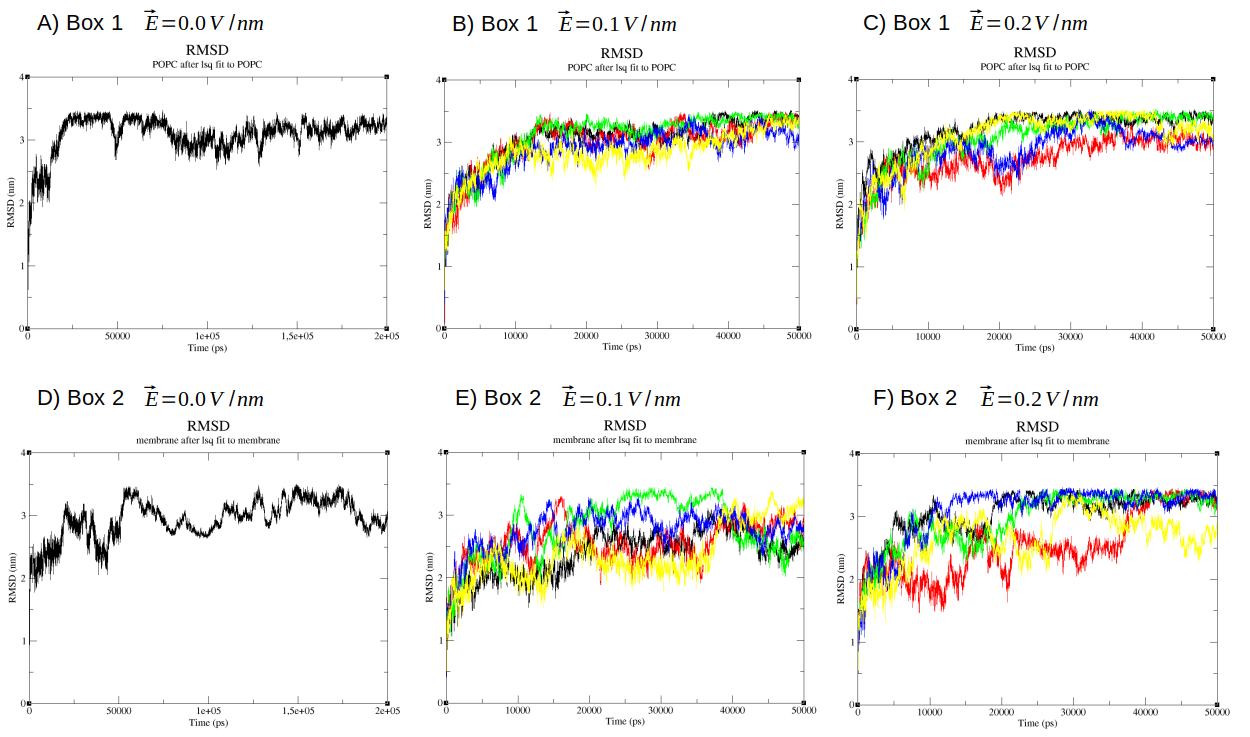

Supplement: Supplementary file 1 [file membranes-11-00473-s001.zip › simulation_controls_1.png]

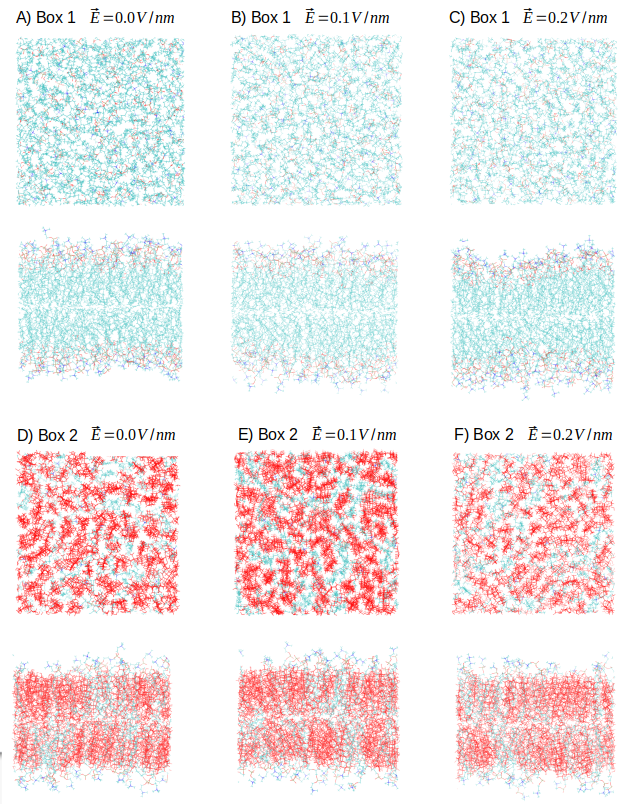

Supplement: Supplementary file 1 [file membranes-11-00473-s001.zip › simulation_controls_2.png]
